# Urban Green Walls under Traffic Stress: Linking the Elemental Composition of Particulate Matter Deposition to Plant Physiological and Gene Expression Responses

**DOI:** 10.1021/acs.est.6c10086

**Published:** 2026-09-18

**Authors:** Lorenzo Massimi, Maria Luisa Antenozio, Davide Marzi, Alice Zara, Caterina Tiraboschi, Federica Porcu, Laura Varone, Silvia Canepari, Tommaso Rossi, Adriana Pietrodangelo, Cinzia Perrino, Patrizia Brunetti

**Affiliations:** † Department of Environmental Biology, 9311 Sapienza University of Rome, P.le A. Moro 5, Rome 00185, Italy; ‡ Research Institute on Terrestrial Ecosystems, 9327 National Research Council of Italy (CNR IRET), Rome 00010, Italy; § National Biodiversity Future Center (NBFC), Palermo 90133, Italy; ∥ Department of Public Health and Infectious Diseases, Sapienza University of Rome, P.le A. Moro 5, Rome 00185, Italy; ⊥ Institute of Atmospheric Pollution Research National Research Council of Italy (CNR IIA), Via Salaria km 29.300, Monterotondo Street, Rome 00015, Italy

**Keywords:** particulate matter, elements, non-exhaust traffic, biomonitoring, plant physiological and gene expression analyses

## Abstract

This one-year study integrates multi-elemental fractionation, physiological, and gene expression analyses to investigate PM retention and plant responses in a nine-species green wall at a high-traffic urban site. A chemical fractionation approach was optimized to discriminate between endogenous and exogenous elements, overcoming a major limitation of conventional methodologies. PCA of the insoluble fraction of Al, Cr, Cs, Fe, Mn, Mo, Rb, Sb, Sn, Ti, and Zn identified *Photinia × fraseri* as the species with the highest and most stable long-term retention of non-exhaust traffic-derived particles, whereas *Pteris vittata* showed the lowest and most unstable retention capacity. Insoluble elements reliably traced PM deposition, while soluble and leaf fractions were strongly influenced by endogenous pools of both essential nutrients and tracers such as Cs and Rb, potentially leading to overestimation of PM deposition by conventional approaches. SEM–EDS confirmed up to 100-fold higher particle retention in *P. × fraseri* than *P. vittata*. Physiological and gene expression analyses revealed pronounced seasonal modulation in *P. × fraseri*, suggesting greater physiological and molecular plasticity in response to environmental fluctuations, whereas *P. vittata* maintained comparatively stable photosynthetic and transcriptional profiles. The results provide a robust framework for plant-based biomonitoring and urban green infrastructure design.

## Graphical abstract

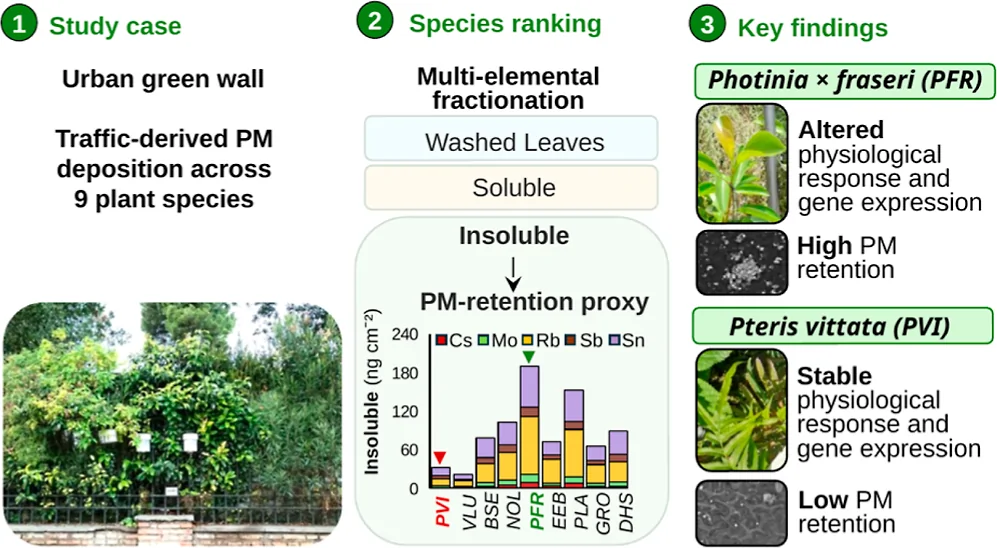

## 1 Introduction

Urbanization has driven profound environmental changes, including biodiversity loss, climate change, and, notably, deteriorating air quality, particularly in densely populated areas with heavy vehicular traffic.^1^ Among atmospheric pollutants, particulate matter (PM) is of major concern due to its well-documented impacts on human health and ecosystems. Traffic, including non-exhaust emissions (NEEs) such as brake and tire wear as well as the resuspension of soil and road dust, represents a significant source of urban PM and potentially toxic elements (PTEs).^2–5^ These particles are typically enriched in macro-, micro-, and trace elements such as Al, Cr, Cs, Fe, Mn, Mo, Rb, Sb, Sn, Ti, and Zn, which serve as reliable tracers of non-exhaust vehicular emissions.^6–8^ Nature-based Solutions (NbS), including green infrastructure (GI), are increasingly recognized for their potential to improve air quality while simultaneously providing cost-effective biomonitoring of air pollution, in urban areas often providing a higher spatial resolution than conventional monitoring networks.^9–12^ At the same time, these systems can operate actively, by forcing air through vegetated substrates, or passively, through particle deposition onto leaf surfaces via gravitational settling and diffusion.^13–15^ Passive deposition onto foliage represents a key mechanism governing the interaction between vegetation and atmospheric particles.^16^

Numerous studies have demonstrated the suitability of plant species for tracking PM pollution from a variety of sources, including industrial emissions, biomass burning, and traffic-related activities.^17–19^ Beyond pollutant accumulation, plant-based biomonitoring provides insight into biological responses to atmospheric contamination.^20^ Non-destructive techniques including chlorophyll fluorescence and leaf spectral reflectance measurements, are sensitive indicators of pollutant-induced alterations in photosynthetic and oxidative stress responses.^21,22^

PM deposition on leaf surfaces is strongly influenced by both environmental conditions and species-specific leaf traits, such as surface roughness, trichomes, epicuticular waxes, stomatal density, and leaf geometry.^23–27^ Despite the growing body of literature, the species-specific mechanisms governing PM deposition and retention remain poorly understood.^28,29^ Likewise, most molecular studies have focused on gaseous pollutants under laboratory conditions,^30,31^ whereas gene expression responses to PM under real-world conditions have only been investigated in a few species, including *Laurus nobilis* and *Photinia × fraseri*, and are restricted to short-term experiments on potted plants.^32,33^

As a result, significant knowledge gaps persist regarding the effectiveness of different species in capturing urban PM and PTEs derived from NEEs, as well as the role of leaf functional traits in modulating these processes.

A major limitation of the chemical methodologies commonly used to quantify elements associated with PM deposited on leaf surfaces relies on the use of the whole-leaf extracts.^34–36^ This approach may lead to overestimations of PM deposition, since water-soluble elements absorbed by roots from soil or fertilizers, can be translocated to leaves, and subsequently released on leaf surfaces through physiological processes, such as evapotranspiration. Consequently, conventional whole-leaf analyses cannot distinguish internally accumulated elements from those derived from surface-deposited PM. Separating insoluble and water-soluble fractions of leaf-deposited PM enables a more reliable identification of PM tracers and provides a more accurate assessment of PM deposition and plant retention potential.^19,37,38^

Beyond particle retention, plants have evolved physiological and molecular mechanisms to cope with air pollution, yet studies integrating these responses with the chemical characterization of leaf-deposited PM under field conditions remain scarce. Such an integrated approach is essential to improve plant-based biomonitoring and optimize urban green infrastructures.

To address this gap, a one-year monitoring study was conducted within the PNRR project *National Biodiversity Future Center* using a nine-species green wall (Figure 1, Table S1) installed at a high-traffic site in Rome (Italy). A chemical fractionation procedure^19,39^ was applied to quantify 25 macro-, micro-, and trace elements in the insoluble, water-soluble, and leaf fractions, enabling the discrimination of atmospheric deposition from physiological accumulation. PM composition and leaf particle distribution were further characterized by SEM–EDS, while physiological traits and gene expression analyses were integrated to identify species-specific responses and evaluate their suitability for urban PM biomonitoring.

**Figure 1:**
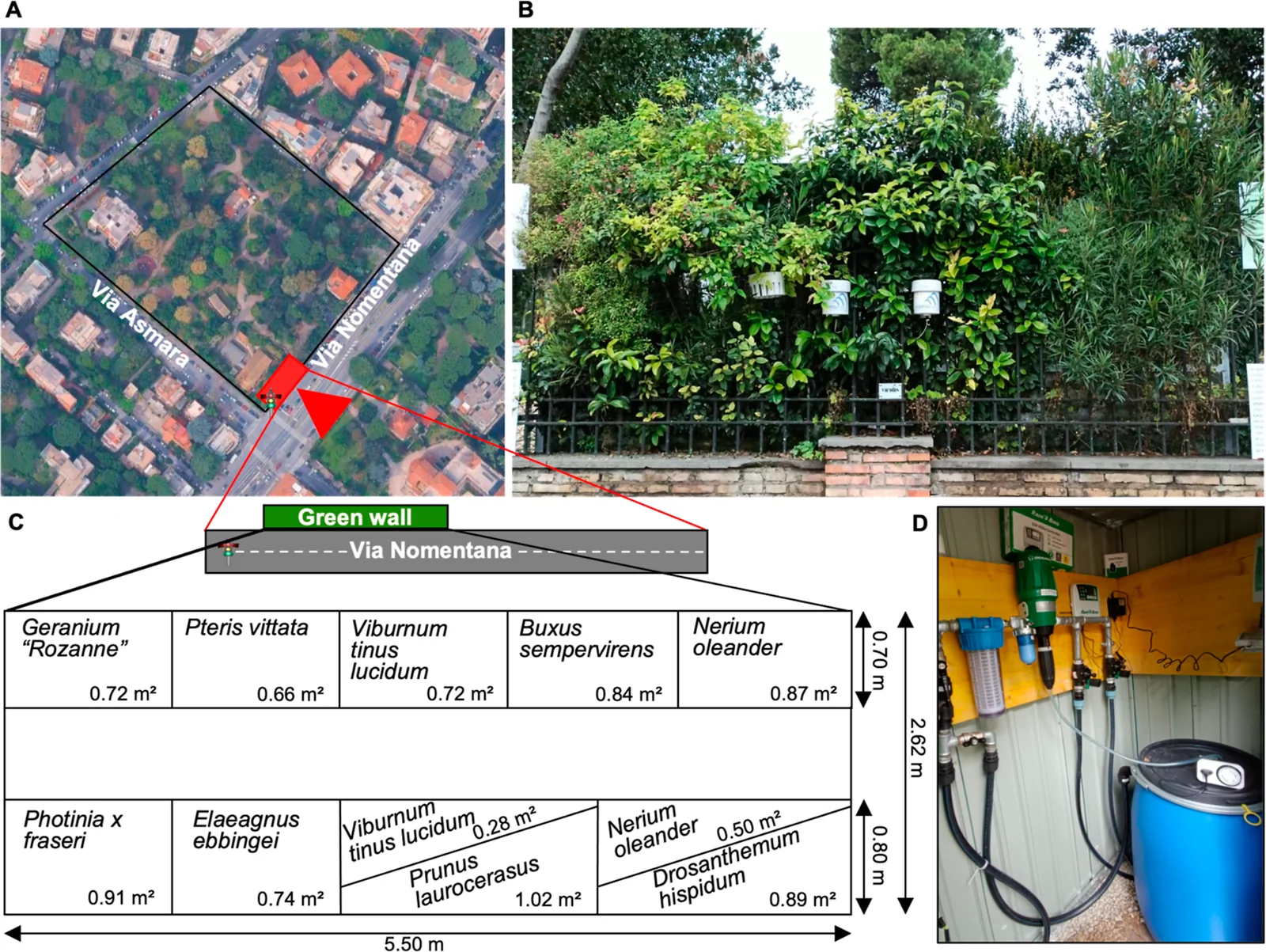
Overview of the study. (A) Location of the green wall in the urban area of Rome, Italy­(via Nomentana inside Villa Leopardi). (B) View of the green wall. (C) Zoom of the “site plan” of the green wall design with the name and location of the nine plant species. (D) Fertigation system to ensure a continuous water and nutrient supply to the green wall.

## 2 Materials and Methods

### 2.1 Study Area, Green Wall Structure, and Experimental Setup

The study was conducted at a high-traffic site in the urban area of Rome (Italy), along Via Nomentana, at the intersection with Via Asmara (41°55′34.3″N 12°31′19.2″E, Figure 1A), characterized by heavy vehicular traffic, high urban density, and frequent stop-and-go conditions due to traffic lights and a nearby bus stop. These features make traffic the dominant local source and promote non-exhaust emissions, such as brake and tire wear, as well as soil and road dust resuspension.

The green wall was an artificially irrigated 5.50 × 2.62 m (length × height) waterproof steel module produced by Viridis Design (Rome, Italy), consisting of two metal panels (5.50 × 0.70 and 5.50 × 0.80 m) separated by a ventilation gap to ensure adequate air circulation around the plants (Figure 1B–D). Both panels were coated with a felt layer equipped with planting pockets hosting nine plant species.

### 2.2 Experimental Workflow and Leaf Sampling Strategy

The experimental workflow is summarized in Figure S1. Following an initial acclimation period after the installation of the green wall, leaf sampling was conducted monthly from July to December 2023 on all nine plant species to quantify the water-soluble, insoluble, and endogenous elemental fractions and rank species according to their PM retention efficiency. In parallel, physiological measurements and leaf sampling for gene expression analyses were performed every two months on all species (Table S2). Based on the elemental analysis performed during this first monitoring phase, *Photinia × fraseri* and *Pteris vittata*, representing the highest and lowest PM-retaining species, respectively, were selected for further investigation.

From January to July 2024, these two species were sampled every two months for chemical fractionation (insoluble, soluble, and endogenous leaf fractions), SEM–EDS microanalysis, physiological measurements, and gene expression analyses, enabling the direct integration of chemical, physiological, and molecular datasets under real urban conditions (Supporting Information 1.1). The bimonthly sampling interval was selected to capture biologically meaningful physiological and molecular adjustments while minimizing the influence of short-term environmental variability.

To ensure comparable exposure conditions and minimize wash-off effects, only mature leaves were collected, whereas young leaves were excluded. All sampling campaigns were performed after at least seven consecutive rain-free days.

### 2.3 Chemical Sample Processing and Elemental Analysis

For each species, at least five mature leaves were collected. Leaves were handled exclusively by the petiole to avoid contamination of the leaf surface. Samples were individually wrapped in clean baking paper, labelled, and stored in polyethylene bags.

For each species and sampling date (Table S2), one representative leaf was selected, placed on graph paper, photographed, and analyzed using a calibrated reference scale. Leaf area (Supporting Information 1.2, Figure S2) was estimated using ImageJ software.^40^ The same leaf was then weighed (fresh weight) and oven-dried (Binder Drying oven, model FED 056, Merck, Darmstadt, Germany) at 60 °C for 24 h to determine dry weight. The fresh weight-to-leaf area and dry weight-to-leaf area ratios obtained from these representative leaves were used to estimate the total leaf area of the analytical samples through proportional calculations. This procedure was repeated for each sampling period to account for seasonal variations in leaf water content and tissue composition, which could affect the fresh weight-to-leaf area relationship. Subsequently, for each species and sampling date, all five mature leaves per plant (approximately 1 g of fresh leaf material ensuring adequate dust water extraction) were subjected to chemical fractionation and elemental analysis.

The actual fresh weight of each sample was recorded, and the corresponding leaf area was calculated using the proportion

$$A_{rl}\left({cm}^{2}\right):{FW}_{rl}\left(g\right)=A_{s}\left({cm}^{2}\right):{FW}_{s}\left(g\right)$$

where *A*_rl_ is the leaf area of the representative leaf in cm^2^; FW_rl_ is the fresh weight of the representative leaf in grams; *A*_s_ is the estimated sample leaf area in cm^2^ and FW_s_ is the fresh weight of the analytical sample (g).

The same proportional approach was applied to dry weight measurements to estimate leaf area for the dried leaf samples.

Subsequently, to separate the different fractions of PM deposited on leaf surfaces, a chemical fractionation procedure adapted from Canepari et al.,^39^ was applied.^19^ For each sample, approximately 1 g of fresh leaves was placed in a 50 mL polypropylene tube and washed with 10 mL of ultrapure water under rotary agitation for 1 h. The washing solution was then filtered through 0.22 μm cellulose nitrate membrane filters (MF-Millipore), allowing the separation of the water-soluble fraction (filtrate) and the insoluble fraction (residue) retained on the filter membrane. The filter membrane with the residue, corresponding to the insoluble fraction of PM deposited on the leaf surface, was subjected to microwave-assisted acid digestion (Ethos Touch Control, Milestone, Italy) using a mixture of HNO_3_ (2 mL; 65%, analytical grade) and H_2_O_2_ (1 mL; Suprapur grade) under controlled conditions. After digestion, the solutions were filtered through 0.45 μm syringe filters (nitrate membrane filters; MF-Millipore) and diluted to a final volume of 50 mL with ultrapure water. This fractionation approach enables the identification of two distinct fractions of PM deposited on the leaf surface, discriminating between the water-soluble fraction, generally considered more bioavailable and potentially more reactive, and the insoluble fraction, typically associated with less bioavailable particulate components.

The washed leaves retrieved after the filtration step were then oven-dried (Binder Drying oven, model FED 056, Merck, Darmstadt, Germany) at 60 °C for 24 h, homogenized using a ball mill (Powteq Micro Ball Mill GT300, Beijing Grinder Instrument Co., Ltd., Beijing, China) at 800 rpm for 20 min, and a subsample (0.1 g) was subjected to microwave-assisted acid digestion using the same procedure described above. This fraction, hereafter referred to as the “leaf” fraction, represents elements retained within or strongly associated with leaf tissues after washing.

All three fractions (leaf, water-soluble, and insoluble deposited PM) were analyzed by inductively coupled plasma mass spectrometry (ICP–MS; PlasmaQuant MS Q, Analytik Jena, Germany) for the determination of macro-, micro- and trace elements. A total of 25 elements were quantified: Al, As, Be, Bi, Cr, Cs, Fe, K, La, Li, Mg, Mn, Mo, Na, Ni, Pb, Rb, Sb, Sn, Ti, Tl, U, V, Zn, and Zr (Tables S3–S5). The same elements were analysed in the fertilizer and in the fertigation solution used in the green wall after filtration through 0.22 μm cellulose nitrate membrane filters (MF-Millipore) and proper dilution of the samples (Table S6). Although Cu is recognized as a selective tracer of non-exhaust traffic emissions, it was not analysed in this study due to its very high amount in the fertilizer, which led to misestimation of its environmental concentration.

Particular attention was given to Al, Cr, Cs, Fe, Mn, Mo, Rb, Sb, Sn, Ti, and Zn, selected as elements associated with non-exhaust traffic emissions, including mineral and resuspended road dust and brake and tire wear. Among these, Cr, Fe, Mn, Mo, Sb, Sn, Ti, and Zn have been widely used as tracers of traffic-related wear processes, although their source specificity varies among elements.^6,8,17,41–44^ External calibration was performed using multi-element standard solutions (1000 ± 2 mg L^–1^), prepared by serial dilution in the same acid matrix as the samples. Yttrium (Y) was used as an internal standard to correct for instrumental drift, signal instability, and matrix effects. The internal standard was continuously introduced online and monitored throughout the analytical sequence.

Limits of detection (LODs) for each element (Table S6) were calculated as the mean concentration of 10 method blank measurements plus three times the standard deviation (SD). Precision was assessed through replicate analyses, with relative standard deviations (RSDs) below 20% for all quantified elements (Table S6), demonstrating the high analytical quality and reproducibility of the dataset.

Thanks to the leaf area-to-weight conversion procedure described above, elemental concentrations could be expressed either on a leaf surface area basis (ng cm^–2^) or on a mass basis (ng g^–1^). In this study, elemental concentrations associated with the water-soluble and insoluble PM fractions deposited on leaf surfaces were preferentially expressed as ng cm^–2^, as surface-area normalization is more appropriate for describing particle deposition processes and enables more reliable comparisons among species with different leaf morphological and structural traits. Indeed, expressing deposited PM on a mass basis may introduce biases related to interspecific differences in leaf thickness, density, and biomass. Conversely, elemental concentrations measured in the leaf tissue fraction were expressed as ng g^–1^, since mass-based normalization better reflects elemental accumulation within leaf tissues. Nevertheless, whenever direct comparisons among the three fractions were required, the proportional conversion derived from the equation described above was applied to express all data using the same unit of measurement.

### 2.4 Multivariate Statistical Analysis

Explorative principal component analysis (PCA) was performed to investigate the main sources of variability within the dataset and to explore relationships among plant species, sampling periods, and elemental concentrations measured in the three fractions: water-soluble (s), insoluble (i), and leaf tissue (leaf).

The analysis was conducted on a total of 54 samples, corresponding to nine plant species collected over 6 consecutive sampling periods from July to December 2023. Subsequent sampling periods were not included in the PCA, as the multivariate analysis was specifically aimed at identifying the plant species showing the most contrasting interactions with elements in deposited PM. The PCA results were therefore used as a screening tool to select the species for further chemical, physiological, and molecular analyses. These subsequent datasets were excluded from the multivariate analysis to maintain homogeneity in sample number and dataset structure among species within the PCA dataset.

A total of 75 variables were included in the analysis, corresponding to the concentrations of 25 elements (Al, As, Be, Bi, Cr, Cs, Fe, K, La, Li, Mg, Mn, Mo, Na, Ni, Pb, Rb, Sb, Sn, Ti, Tl, U, V, Zn, and Zr) measured in each of the three fractions (Tables S3–S5). The resulting dataset provided an integrated description of elemental partitioning between surface-deposited PM fractions and leaf-retained elemental components.

Before PCA, the data matrix was pre-treated by column mean centering and autoscaling (unit variance scaling) to minimize the influence of differences in concentration magnitude and variability among variables. PCA was then performed on the standardized dataset to reduce dimensionality and identify the principal sources of variance within the system. Multivariate statistical analyses were performed using the CAT tool (Leardi at al.: Chemometric Agile Tool, freely downloadable from http://gruppochemiometria.it/index.php/software) based on R software (R Foundation for Statistical Computing, Vienna, Austria).

### 2.5 SEM–EDS Microanalysis

Representative leaf portions of *P. × fraseri* and *P. vittata* were mounted on carbon-coated SEM stubs and analysed by scanning electron microscopy (SEM; Quanta 250F FEG SEM, Thermofisher Scientific) under high-vacuum conditions to characterize particles deposited on leaf surfaces. Particle morphology and elemental composition were investigated through SEM coupled with energy-dispersive X-ray spectroscopy (SEM-EDS). Backscattered electron imaging (BSE) was used to identify particles with higher atomic number contrast relative to the leaf substrate, allowing selective analysis of mineral and metal-rich particles typically associated with traffic-related non-exhaust emissions. This approach exploits the low average atomic number of leaf tissues, which enhances the detection of silicate and metallic particles.^45,46^

Automated single-particle analysis was performed on selected leaf areas by a EDAX Octane Elect Plus (30 mm^2^) silicon drift detector (SDD), using the Particle Phase Analysis Genesis v. 5.10 package (Ametek Inc., 2006), which simultaneously provided particle size, morphology, and elemental composition. Between 700 and 1000 particles were analysed per sample. Elemental concentrations were quantified by ZAF weight percent (% wt) method of the Genesis software. The method determines the element weight concentrations in the electron interaction volume, by calculating the element k-ratios relative to standards (or estimated standards in standardless mode) under Castaing’s first approximation corrected for bulk matrix effects (atomic number, absorption, fluorescence). Particle masses were estimated from elemental composition, oxide densities, and particle volume, assuming spherical geometry.^47,48^ The cumulative mass of each element was then normalized to the analysed leaf surface area (ng cm^–2^). Particle classes were obtained by decreasing ranking element abundances on particle-by-particle basis.^49^

### 2.6 Photosynthetic Performance and Leaf Reflectance Measurements

Photosynthetic performance was assessed by measuring the effective quantum yield (ΦPSII) and maximal quantum yield (Fv/Fm) of PSII directly on the plant species of the green wall using a handheld fluorometer (FluorPen FP 110, Photon Systems Instruments, Drásov, CR) between 10:00 and 10:30 am. Two measurements were carried out on five different leaves per plant, for a total of ten measurements.

ΦPSII was calculated on light-adapted leaves according to^50^ as ΦPSII = (*F*_m_′ – *F*_s_)/*F*_m_′, where *F*_m_′ is the maximum fluorescence induced by a saturating pulse (8000 μmol photons m^–2^ s^–1^ and *F*_s_) is the steady-state fluorescence measured under actinic light (1600 μmol photons m^–2^ s^–1^). *F*_v_/*F*_m_ was measured on leaves, which were dark-adapted for 30 min. After this period, the minimum fluorescence (*F*_0_) was measured using a weak modulated measuring beam, followed by a saturating red-light pulse (8000 μmol m^–2^ s^–1^) to determine the maximum fluorescence (*F*_m_); *F*_v_/*F*_m_ was calculated as (*F*_m_ – *F*_0_)/*F*_m_.

Reflectance indices were obtained from the same individual leaves used for chlorophyll fluorescence measurements for each plant on each measurement day, using a radiometrically calibrated handheld spectroradiometer (SpectraPen LM 510-H/UVIS, Photon Systems Instruments, Drásov, CR) with a wavelength range of 340–790 nm. At least two measurements for each collected leaf were conducted immediately after chlorophyll fluorescence measurements, between 10:30 and 11:00 am. After calibrating the instrument with a standard calibration plate (Photon Systems Instruments, Drásov, CR), the leaves were placed directly in the leaf compartment of the instrument, with the adaxial side of the leaf facing the sensor. Specific wavelengths obtained from the reflectance spectra were used to calculate various vegetation indices, such as the normalized difference vegetation index (NDVI), calculated as NDVI = (R780 – R630)/(R780 + R630), the photochemical reflectance index (PRI), calculated as PRI = (R531 – R570)/(R531 + R570), the carotenoid reflectance indices (CR1) as CR1 = (1/R510) – (1/R550), and the anthocyanin reflectance indices (ARI1) as ARI1 = (1/R 550) – (1/R700), where R represents the reflectance measured at the corresponding wavelength (nm).

### 2.7 RNA Extraction and Quantitative Reverse Transcription PCR Analysis

For gene expression analyses, nine individual plants per species (*P.* *vittata* and *P. × fraseri*) were sampled. One pinna per *P. vittata* and one leaf per *P. × fraseri* were collected from each plant. The samples were subsequently grouped into three independent biological replicates (*n* = 3). All samples were immediately frozen in dry ice and 95% ethanol and stored at −80 °C until analysis. Frozen samples were ground in liquid nitrogen, and total RNA was extracted from each pool using NORGEN—Plant/Fungi Total RNA Purification kit. RNA quantity and quality were assessed by spectrophotometry (NanoDrop, Thermo Fisher Scientific Inc, USA) and agarose gel electrophoresis.

cDNA synthesis was performed using PrimeScriptTM RT Reagent Kit with gDNA Eraser (TAKARA). Gene expression analysis was carried out by SYBR Green-based quantitative reverse transcription PCR (RT-qPCR) as previously described.^51^Relative expression levels of the *P. vittata* genes encoding superoxide dismutase (*PvSOD*), ascorbate peroxidase (*PvAPX*), cellulose synthase A (*PvCesA*), and β-galactosidase 8 (*PvβGal8*) were normalized against elongation factor 1-β (*PvEF1b*). Similarly, the expression levels of the *P. × fraseri* genes encoding cellulose synthase (*PfCesA*), ascorbate peroxidase 2 (*PfAPX2*), iron superoxide dismutase (*PfFeSOD*), and β-galactosidase 2 (*PfβGal2*) were normalized against formate–tetrahydrofolate ligase (*PfTHF*). Primer sequences are reported in Table S7. Each biological replicate quantification was performed in technical triplicates. Data analysis was performed using the 2^–Δ*Ct*^ method.^52,53^

### 2.8 Statistical Analysis

To evaluate the temporal stability of elemental concentrations, descriptive statistics were calculated for each element, species, and fraction over the monitoring period. The coefficient of variation (CV %) was used as an indicator of central tendency and temporal variability. The mean was calculated as the average concentration across all sampling dates, whereas the standard deviation was used to quantify the dispersion of values around the mean. Temporal variability was assessed using the coefficient of variation, calculated as

$$Mean=\Sigma x_{i}/n$$

$$SD=\surd \left[\Sigma {\left(x_{i}-\mathrm{x̅}\right)}^{2}/\left(n-1\right)\right]$$

CV(%)=(SD/mean)×100

Physiological and gene expression data normality and homogeneity of variances were assessed using the Shapiro–Wilk and Levene’s tests, respectively. Relative gene expression data were analysed separately for each species by one-way analysis of variance (ANOVA), considering sampling time as the fixed factor.

Physiological data outliers were identified using the interquartile range (IQR) method. Differences among species and sampling dates were evaluated by two-way ANOVA, considering species and sampling time as fixed factors.

When significant differences were detected (*P* < 0.05), pairwise comparisons among sampling dates were performed using Tukey’s HSD post hoc test. Data are presented as mean ± SD and different letters indicate statistically significant differences among sampling dates at *P* < 0.05.

## 3 Results and Discussion

### 3.1 Explorative Principal Component Analysis

Explorative PCA was applied to the complete dataset collected from July 2023 to December 2023, including all plant species and elemental concentrations (Tables S3–S5) measured in the three operationally defined fractions: leaf tissue (leaf), water-soluble (s) and insoluble (i).

The PCA score plot revealed a distinct species-dependent clustering of samples, demonstrating that plant-specific traits played a key role in determining elemental composition and fraction partitioning (Figure 2). Samples belonging to the same species generally clustered together regardless of sampling period, indicating that interspecific differences accounted for a substantial proportion of the observed variability. These findings suggest that species-specific morphological and functional characteristics largely control the interaction between deposited PM and leaf surfaces.

**Figure 2:**
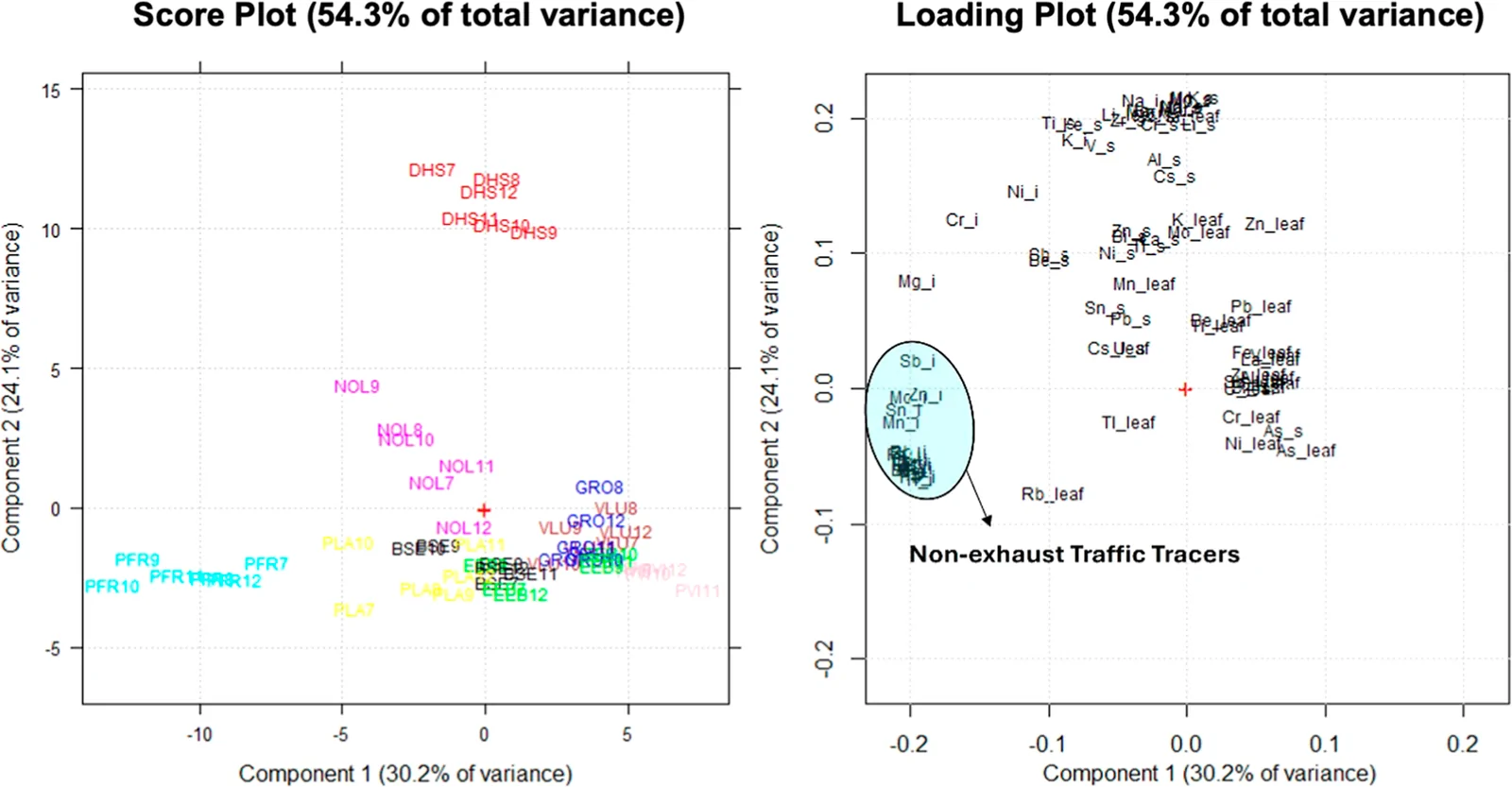
Principal component analysis (PCA) of elemental concentrations measured in the insoluble (i), soluble (s), and leaf fractions across all the investigated plant species. The score plot (left) shows the distribution of samples according to the first two principal components, which together explain 54.3% of the total variance (PC1 = 30.2%, PC2 = 24.1%). The loading plot (right) illustrates the contribution of individual elements and fractions to sample separation. Sample labels consist of a species acronym followed by the sampling month: BS (black) = *Buxus sempervirens*, DHS (red) = *Drosanthemum hispidum*, EEB (green) = *Elaeagnus ebbingei*, GRO (blue) = *Geranium “Rozanne”*, NOL (magenta) = *Nerium oleander*, PFR (cyan) = *Photinia × fraseri*, PLA (yellow) = *Prunus laurocerasus*, PVI (light pink) = *Pteris vittata*, and VLU (dark red) = *Viburnum lucidum*. The number associated with each sample indicates the month of leaf sampling and chemical analysis (7 = July, 8 = August, 9 = September, 10 = October, 11 = November, and 12 = December).

PC1 represented the main gradient separating samples with higher concentrations of insoluble elements in deposited PM from those with higher concentrations of water-soluble and leaf-retained elements. In the loading plot, several insoluble elements, including PTEs such as Al, Cr, Cs, Fe, Mn, Mo, Rb, Sb, Sn, Ti, and Zn, clustered on the negative side of PC1, within the region identified as non-exhaust traffic tracers, suggesting that negative PC1 scores were mainly associated with the retention of insoluble particles deposited on leaf surfaces. This fraction mainly represents particles physically deposited on leaf surfaces and is therefore most closely associated with NEEs, including brake and tire wear as well as road dust resuspension. Conversely, many water-soluble and leaf-retained elements were distributed toward the positive side of PC1, indicating a different behavior of elements occurring in more mobile or leaf-associated fractions. Among the investigated species, *Photinia × fraseri* showed the strongest shift toward negative PC1 values. Its samples clustered close to the group of insoluble elements associated with non-exhaust traffic tracers, indicating a pronounced retention of insoluble PM components from vehicular traffic. In contrast, *P. vittata* samples were located on the positive side of PC1 and were clearly separated from the cluster of traffic tracers, indicating a weaker association with insoluble deposited PM. The clear separation between *P. × fraseri* and *P. vittata* highlights the usefulness of PCA as a screening tool to identify species with contrasting interactions with deposited PM, supporting their selection for further physiological and molecular analyses.

PC2 further contributed to the separation among species and elemental fractions. *Drosanthemum hispidum* was clearly separated along the positive side of PC2, indicating a distinct elemental profile compared with the other species. Its position suggests that this species was mainly associated with water-soluble and leaf-retained elements located in the upper part of the loading plot. *Nerium oleander* also showed positive PC2 scores, although with a less extreme separation than *D. hispidum*, suggesting an intermediate but still distinctive behaviour. In contrast, several species, including *Buxus sempervirens*, *Elaeagnus ebbingei*, *Geranium “Rozanne”*, *Prunus laurocerasus*, and *Viburnum lucidum*, clustered closer to the centre or toward positive PC1 values, indicating fewer associations with the insoluble elements in PM depositions.

The loading plot highlighted a clear organization of variables according to elemental fraction. Insoluble elements associated with non-exhaust traffic PM were grouped in the negative PC1 region, whereas water-soluble and leaf-retained elements were more widely distributed from the central to positive PC1 regions. The proximity between several water-soluble and leaf-retained elements suggests a close relationship between the most bioaccessible elemental forms and the elements retained within or strongly associated with leaf tissues. This pattern indicates that the water-soluble fraction, representing the most environmentally mobile and potentially bioaccessible PM component, may be more actively involved in leaf–particle interactions, uptake, or retention processes, whereas insoluble fractions mainly reflect the physical retention of deposited particles on the leaf surface. The stronger correspondence observed between water-soluble elements deposited on leaf surfaces and the elemental composition of leaf tissues may also plausibly reflect physiological processes unrelated to direct PM deposition. In particular, elements previously absorbed by the root system and subsequently translocated to the leaves may be released through evapotranspiration during warmer periods and later detected as soluble residues on the leaf surface. Consequently, part of the soluble fraction may derive not only from atmospheric PM deposition but also from endogenous plant processes. This interpretation further supports the importance of the adopted chemical fractionation approach, which enables a clearer separation between deposited PM and leaf-associated elemental components.

### 3.2 Temporal Trends of Selected Insoluble Elemental Tracers of PM Deposition from Non-Exhaust Traffic Emissions

The monitoring campaign encompassed both summer and winter conditions, enabling the assessment of PM retention dynamics under progressively evolving deposition patterns over time (Supporting Information 1.3, Table S2). To focus on the most relevant traffic-related signatures, 11 (Al, Cr, Cs, Fe, Mn, Mo, Rb, Sb, Sn, Ti, and Zn) out of the 25 measured elements were selected. The quantitative evaluation of elemental deposition patterns further supports the contrasting PM–leaf interaction behaviors identified by PCA.

A common temporal trend was observed for all classes of insoluble elements (i.e., macro-, micro-, and trace elements), with concentrations increasing from July to October as a result of progressive accumulation over time and declining thereafter (Figure 3A–C). This pattern likely reflects seasonal meteorological factors, as the warmer and drier conditions favor road dust resuspension and consequently leaf deposition of soil-derived and non-exhaust traffic PM,^44^ whereas the higher rainfall frequency from November onwards reduces both particle resuspension and leaf surface accumulation through wash-off.

**Figure 3:**
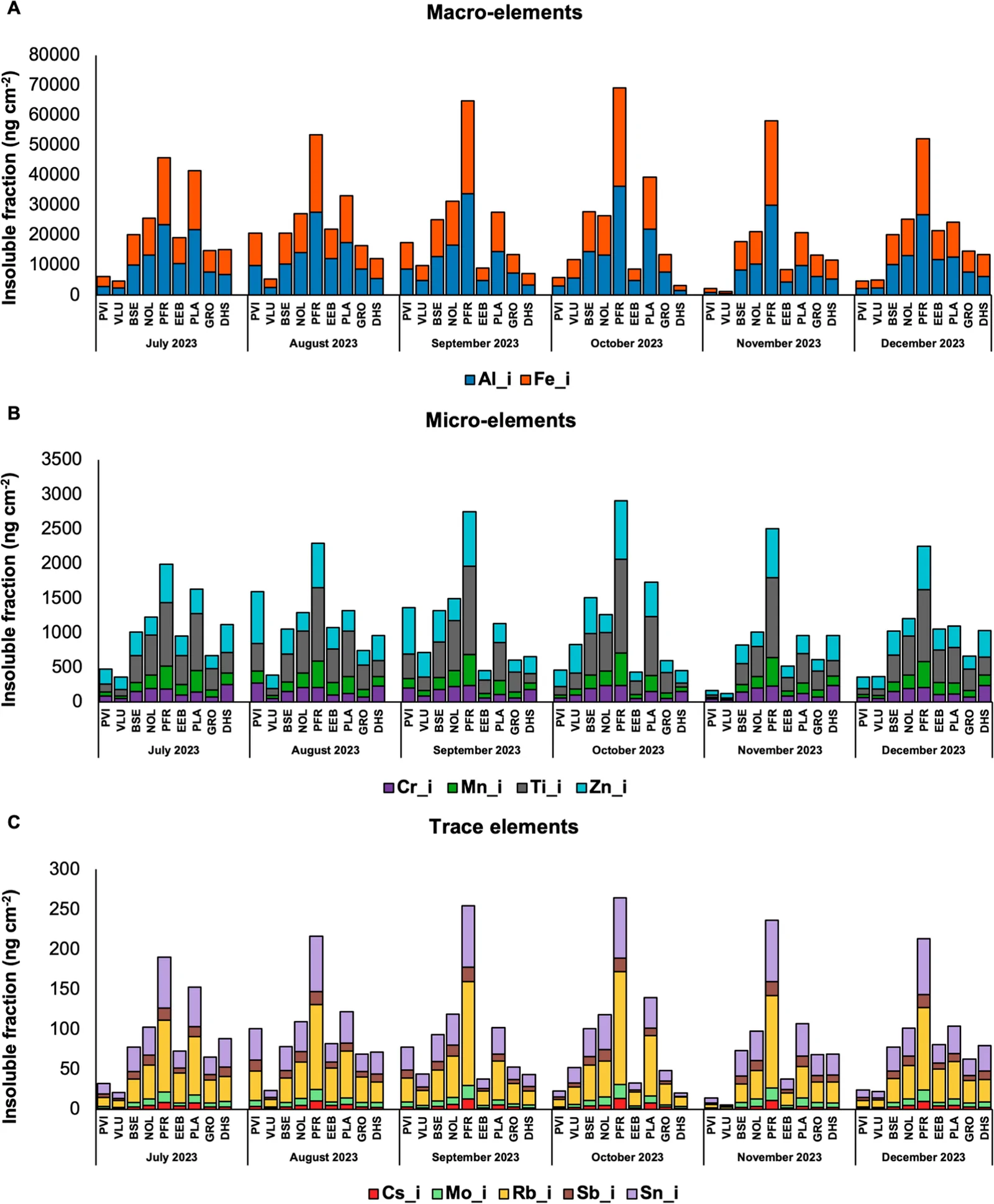
Stacked bar charts showing the insoluble elemental fraction of PM deposited on leaf surfaces, grouped into (A) macro-elements (≤40,000 ng cm^–2^), (B) micro-elements (≤1500 ng cm^–2^), and (C) trace elements (≤150 ng cm^–2^). Sample labels consist of a species acronym followed by the sampling month: BS = *Buxus sempervirens*, DHS = *Drosanthemum hispidum*, EEB = *Elaeagnus ebbingei*, GRO = *Geranium “Rozanne”*, NOL = *Nerium oleander*, PFR = *Photinia × fraseri*, PLA = *Prunus laurocerasus*, PVI = *Pteris vittata*, and VLU = *Viburnum lucidum*.

Across all species, Al and Fe were the major elements, with maximum concentrations reaching approximately 37,000 ng cm^–2^ (Figure 3A). Insoluble micro-elements, including Cr, Mn, Ti, and Zn, ranged from about 250 to 1400 ng cm^–2^ (Figure 3B), whereas trace elements such as Cs, Mo, Rb, Sb, and Sn generally remained below 150 ng cm^–2^ (Figure 3C). Consistent with the PCA results (Figure 2), *P. × fraseri*, followed by *P. laurocerasus* and *N. oleander*, exhibited the highest concentrations of insoluble elements, indicating a greater capacity to retain firmly adhered particles and providing a stable record of PM deposition over time. Meanwhile, *P. vittata*, *Elaeagnus ebbingei*, and *G. “Rozanne”* consistently displayed the lowest retention efficiency for all elements over time. Notably, *P. vittata* was characterized by an unstable retention trend over the six-month monitoring period. This is probably due to the leaf morphology of these plant species, as shown in Supporting Information 1.2 and Figure S2, and well documented in the literature (Table S1). This efficiency is strongly linked to leaf morphological traits: elliptic, coriaceous leaves with a well-developed cuticle and often rough or waxy surfaces in *P. × fraseri* promote particle adhesion. Conversely, *P. vittata* is characterized by pinnate fronds composed of multiple separate pinnae arranged along a central rachis. These thinner and less coriaceous leaf structures, combined with a less developed cuticle, likely contribute to its lower PM retention capacity (Supporting Information 1.2, Figure S2). Due to its higher efficiency in retaining elements tracing traffic-related sources, as well as the greater availability of gene-level information in the literature, *P. × fraseri* was selected as the model species for integrated chemical, physiological, and molecular analyses. Its suitability as a bioindicator and its well-characterized stress-response pathways make it particularly appropriate for investigating plant responses to urban PM pollution. In contrast, *P. vittata*, which exhibited markedly lower PM retention, was selected as a comparative species to assess how different PM retention capacities influence physiological and molecular responses.

### 3.3 SEM–EDS Characterization of PM Retained on *Photinia × fraseri* and *Pteris vittata* Leaves

SEM microanalysis confirmed the markedly different PM retention capacities of the two selected species, consistent with their different leaf morphology (Figures S2 and S3). The adaxial surface of non-washed leaves of *P. vittata* showed particle abundances on the order of 10^3^ particles cm^–2^, whereas the surface of *P. × fraseri* leaves exhibited much higher densities, on the order of 10^5^ particles cm^–2^. In contrast to *P. × fraseri*, particle abundance on *P. vittata* leaves was further reduced after washing, indicating a very limited capacity to retain deposited particles. Conversely, a substantial number of particles remained attached to *P. × fraseri* leaves after washing, with particle density decreasing from approximately 8 × 10^5^ to 5 × 10^5^ particles cm^–2^, highlighting the strong particle retention capacity of its leaf surface (Figure 4).

**Figure 4:**
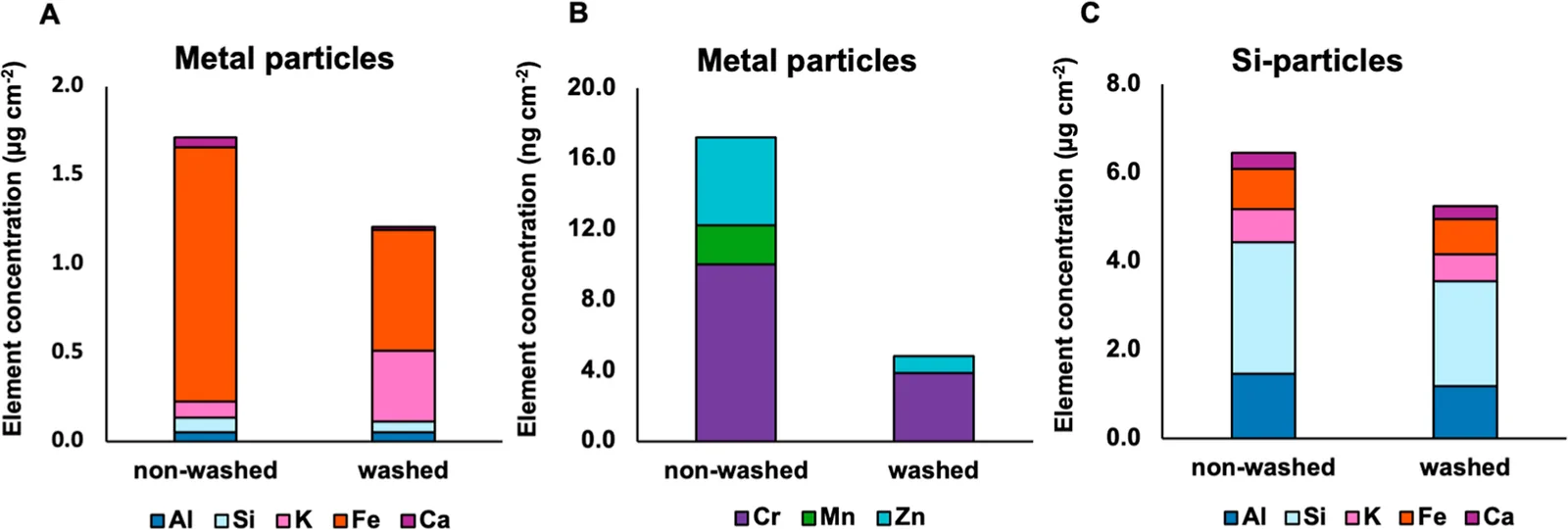
Element concentration attributed to Fe- (Cr/Mn/Zn) and Si-particles in non-washed and washed *P. × fraseri* samples. Macro (μg cm^–2^)- and micro (ng cm^–2^)-elements in metal (A and B respectively) and silicate (C) particles. Based on the 25 keV accelerating voltage used for SEM microanalysis, these data refer to an interaction volume encompassing material from the surface to depths of approximately 10 μm, depending on particle composition and density.

It should be emphasized that the particle abundance estimated from Backscattering Electron (BSE)-SEM images only refers to particles providing sufficient atomic number (Z) contrast relative to the leaf surface to be detected and counted. In fact, particle characterization revealed that the retained PM was mainly composed of silicate (Si-rich), aluminosilicate (Al-rich), and metal (Fe-rich) particles, typically associated with non-exhaust traffic emissions (Figures S4 and 4A). EDS analyses of non-washed *P. × fraseri* leaves showed that metal particles were characterized by high Fe contents, frequently associated with Cr, Mn, and Zn (Figure S4). The numerosity of these particles is about half that of silica and aluminosilicates group (hereafter referred to as silicates) in the non-washed sample, while their abundance decreases to 1:3 with respect to silicates in the washed sample. Fe particles seem to be more efficiently removed by washing than silicates (Figure 4A,B). This could be explained by the influence of the morphological traits of *P. × fraseri* leaf adaxial surface on the retention capacity of different deposited particulate components.^54^ Fe particles show more compact morphology and consequently a small contact area with the leaf surface than silicates, irregularly shaped, this likely causing reduced adhesion of the former to leaf surface.

Silicate and aluminosilicate particles were enriched in Si, Al, K, and Ca, consistent with their origin from crustal materials and resuspended road dust (Figure 4C). Minor amounts of Si, Ca, and K detected in Fe-rich particles are attributable to secondary X-ray fluorescence generated by neighbouring particles during SEM–EDS analysis.^55^

The persistence of a substantial particle load on washed *P. × fraseri* leaves is consistent with the chemical fractionation results and confirms that the insoluble fraction provides the most representative measure of PM-derived non-exhaust traffic tracers retained on leaf surfaces.

### 3.4 Relative Contributions of PM Deposition and Plant Physiological Processes to Elemental Retention

To evaluate the relative contributions of PM deposition and plant physiological processes to elemental retention, mean concentrations measured in the insoluble, soluble, and leaf fractions were compared for selected tracers of non-exhaust traffic-derived PM in *P. × fraseri* and *P. vittata* *.* Chemical fractionation revealed clear differences in the temporal elemental distribution among the three fractions (Tables 1; S8–S10). The insoluble fraction of *P. × fraseri* showed low temporal variability (CV ≈ 22–23%; Max/Min ≈ 2), indicating stable retention of PM-associated elements throughout the monitoring period, whereas *P. vittata* exhibited substantially higher variability (CV ≈ 65–70%), reflecting less consistent particle retention. In contrast, the soluble and leaf fractions were more variable in both species (CV > 30%), indicating a stronger influence of physiological processes than atmospheric deposition (Table 1).

**Table 1:** Temporal Variability of Elemental Concentrations in the Insoluble, Soluble, and Leaf Fractions of *P. × fraseri* and *P. vittata*

species	element	insoluble^a^ (mean ± SD)	CV (%)	soluble^a^ (mean ± SD)	CV (%)	leaf^a^ (mean ± SD)	CV (%)
*P. × fraseri*	Al	31.70 ± 7.11	22	0.02 ± 0.01	51	1.11 ± 0.46	41
	Fe	29.73 ± 6.64	22	0.04 ± 0.01	27	1.89 ± 0.96	51
	K	62.08 ± 13.94	22	205.55 ± 9.18	45	738.16 ± 198.53	27
	Mg	7.05 ± 1.58	22	1.83 ± 0.66	36	54.97 ± 12.61	23
	Cr	240 ± 55	23	1.25 ± 0.39	31	18.48 ± 10.76	58
	Mn	439 ± 98	22	18.01 ± 7.60	42	484 ± 205	42
	Ti	1219 ± 272	22	0.62 ± 0.17	27	85.76 ± 31.75	37
	Zn	744 ± 167	22	79.11 ± 23.72	30	352 ± 98	28
	Cs	11.81 ± 2.65	22	0.19 ± 0.11	57	3.00 ± 0.88	29
	Mo	16.53 ± 3.71	22	0.54 ± 0.21	39	27.90 ± 8.03	29
	Rb	121.83 ± 27.36	22	47.30 ± 25.46	54	458 ± 148	32
	Sb	18.74 ± 4.49	24	0.20 ± 0.05	28	1.85 ± 0.82	44
	Sn	80.08 ± 18.78	23	0.13 ± 0.04	32	3.92 ± 2.38	61
*P. vittata*	Al	4.4 ± 3.02	68	0.01 ± 0.01	90	0.11 ± 0.08	70
	Fe	4.83 ± 3.14	65	<LOD	//	0.29 ± 0.15	52
	K	48.96 ± 30.16	62	85.13 ± 58.30	68	82.65 ± 21.27	26
	Mg	1.87 ± 1.23	62	2.67 ± 1.96	73	4.57 ± 1.62	35
	Cr	123 ± 77	62	0.93 ± 0.75	81	2.14 ± 1.21	56
	Mn	79 ± 50	64	15.49 ± 12.54	81	47.42 ± 27.57	58
	Ti	176 ± 121	69	0.29 ± 0.19	66	6.80 ± 2.76	41
	Zn	332 ± 230	70	82.84 ± 41.25	50	73.14 ± 34.38	47
	Cs	1.56 ± 1.08	69	0.13 ± 0.08	61	0.08 ± 0.03	45
	Mo	3.28 ± 2.10	64	0.80 ± 0.64	80	3.25 ± 1.35	42
	Rb	16.56 ± 10.79	65	31.10 ± 20.10	65	11.31 ± 5.01	44
	Sb	6.17 ± 3.84	62	0.07 ± 0.03	44	0.21 ± 0.14	71
	Sn	17.46 ± 10.87	62	0.09 ± 0.04	48	0.68 ± 0.55	79

a Elemental concentrations are expressed as ng cm^–2^, except for Al, Fe, K and Mg which are expressed as μg cm^–2^. CV percentages were used as an indicator of the temporal stability of elemental concentrations.

Elemental partitioning further highlighted the value of chemical fractionation. Traffic-related tracers such as Al, Ti, Cr, Sb, and Sn were predominantly associated with the insoluble fraction, confirming their predominantly atmospheric origin, while the soluble and leaf fractions accounted for less than 10% of the insoluble pool (Table S9, Leaf/Insol <0.10; Sol/Insol <0.02).

Conversely, Mg, Mn, Mo, K, and Zn showed the highest Leaf/Insol ratios, indicating a strong enrichment in leaf tissues relative to the insoluble fraction (Tables S9 and S10). This pattern is consistent with their physiological roles and root uptake from fertilization (Tables S6–S9).^56,57^ While Mg, Mn, Mo, K and Zn are essential elements involved in key metabolic functions—such as enzyme activation (Mn, Zn), osmotic regulation and stomatal function (K), chlorophyll structure and photosynthesis (Mg), and redox and nitrogen metabolism (Zn, Mo)^56,57^ —it is noteworthy that Rb, and to a lesser extent Cs, despite not being essential nutrients, also exhibited high Leaf/Insol ratio, reaching 3.76 in *P. × fraseri* (Table S10). This enrichment is likely explained by their presence in the fertilizer and subsequent root uptake (Table S6). Their chemical similarity to K probably enables transport through K uptake systems, resulting in their accumulation in leaf tissues, as previously reported.^58,59^ These results demonstrate that total elemental concentrations alone may overestimate PM-derived contributions, whereas chemical fractionation can effectively distinguish deposited particles from endogenous elemental pools.

### 3.5 Physiological Responses Revealed Contrasting Acclimation Strategies in *Photinia × fraseri* and *Pteris vittata*

Chlorophyll fluorescence and spectral indices (Figure 5A–F) revealed contrasting physiological responses that were consistent with the elemental partitioning patterns. Maximum PSII efficiency (*F*_v_/*F*_m_) remained stable in both species (Figure 5A), indicating the absence of severe photoinhibition under urban exposure, consistent with previous observations.^60^ Despite showing the highest and most stable retention of traffic-derived PM (CV ≈ 22%; Table 1), *P. × fraseri* exhibited greater seasonal variability in PRI and NDVI and to a lesser extent ΦPSII (Figure 5B,C). Since PRI and ΦPSII are associated with changes in radiation use efficiency and xanthophyll activity, whereas NDVI reflects variations in leaf greenness and photosynthetically active biomass, these temporal fluctuations may indicate physiological acclimation to changing environmental conditions and to the higher particulate load retained by *P. × fraseri* leaves, rather than a decline in photosynthetic performance. At the same time, the lower pigment-related indices observed in *P. × fraseri* suggest a greater reliance on dynamic physiological regulation rather than constitutively photoprotective pigment pools. The higher accumulation of K and Mg in leaf tissues (Table S10) further supports the maintenance of metabolically active nutrient pools, that may support long-term tissue maintenance and structural investment. In contrast, *P. vittata* maintained relatively stable ΦPSII and PRI together with consistently higher ARI, NDVI, and CRI1 values (Figure 5B–F), despite its lower and more variable retention of insoluble PM. This physiological stability was consistent with the enrichment of Zn, Mn, and Mo in leaf tissues (Table S10), involved in photosynthesis, antioxidant metabolism and stress acclimation, suggesting efficient maintenance of photosynthetic and antioxidant functions. These findings suggest the presence of constitutive photoprotective and antioxidant mechanisms in *P. vittata* that may buffer the physiological effects of fluctuating elemental loads.

**Figure 5:**
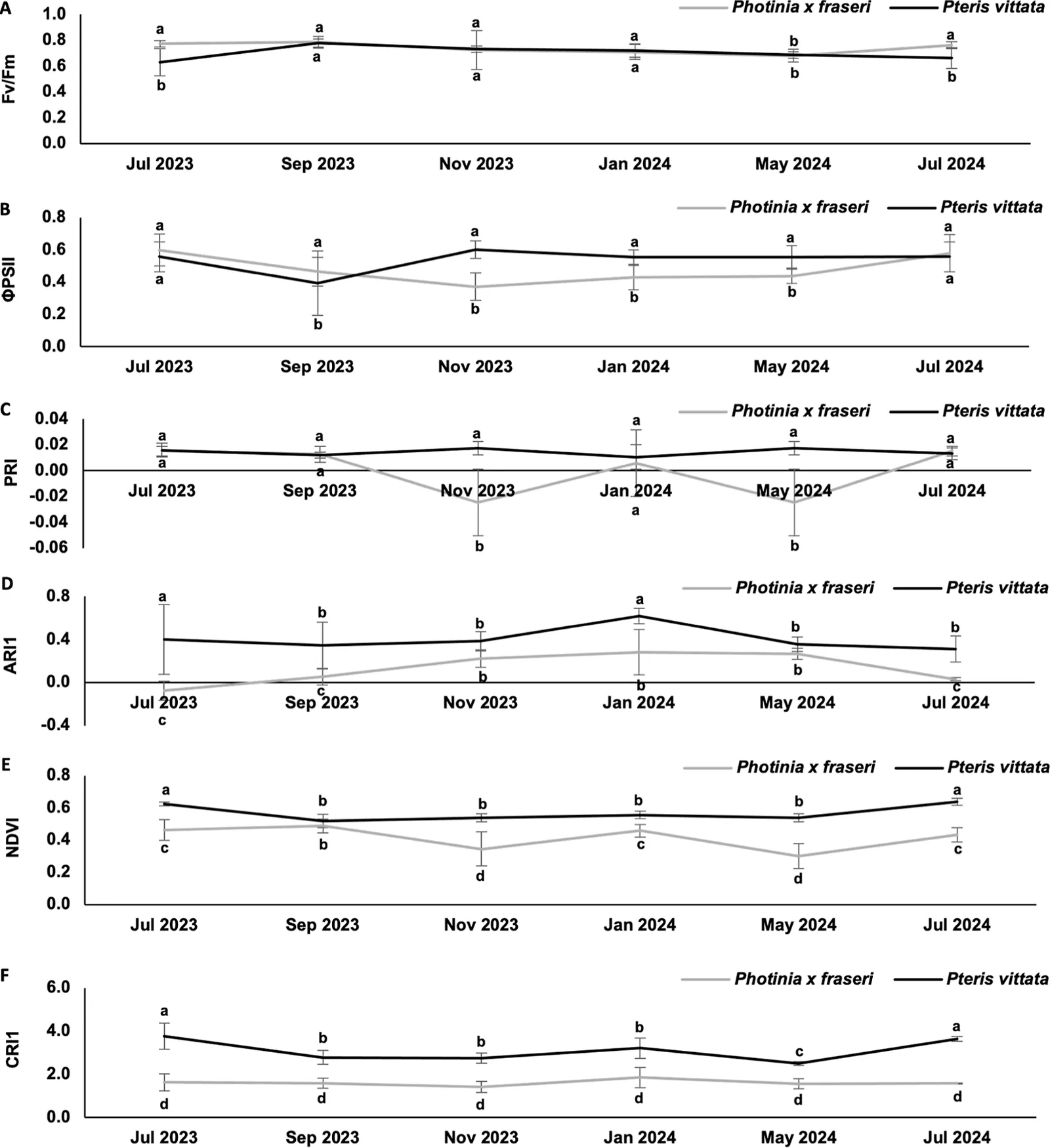
Chlorophyll fluorescence and reflectance indices measured on leaves of *Photinia × fraseri* and *Pteris vittata* from July 2023 to July 2024. (A) Maximal quantum efficiency (*F*_v_/*F*_m_), (B) effective quantum yield (ΦPSII), (C) photochemical reflectance index (PRI), (D) anthocyanin reflectance index (ARI), (E) normalized difference vegetation index (NDVI) and (F) carotenoid reflectance index (CRI1).

### 3.6 Contrasting Gene Expression Responses in*Photinia × fraseri* and *Pteris vittata*

To evaluate the effects of PM deposition on leaf surfaces, the expression of selected stress-related genes involved in antioxidant defence and cell wall modification was analysed.^32,61–63^ The gene expression results are highly consistent with the physiological and elemental data. *P. × fraseri* displayed greater molecular plasticity, with pronounced seasonal regulation of antioxidant- and cell wall-related genes (Figure 6A–D, left panel). *PfFe-SOD* and *PfAPX2* were strongly induced during summer (July 2023), followed by a sharp decrease during autumn and winter and a partial recovery by July 2024 (Figure 6A,B, left panel). Likewise, *PfCesA* exhibited high expression at the beginning of the monitoring period and became nearly undetectable during winter, whereas *Pf*β*Gal2* showed a transient upregulation during spring (Figure 6C,D, left panel). These transcriptional changes were accompanied by fluctuations in Φy and PRI and match with the highest and most stable retention of traffic-related tracers in the insoluble fraction, consistent with a more dynamic acclimation response under sustained urban PM exposure. Conversely, in *P. vittata*, the limited temporal variation in antioxidant- and cell wall-related gene expression (*PvSOD*, *PvAPX* and *PvCesA* and *Pv*β*Gal8* Figure 6A–D, right panel) was associated with the stability of physiological parameters, including photosynthetic efficiency, pigment indices, and antioxidant status, together with the progressive accumulation of Zn and, to a lesser extent, Mn and Mo in leaf fraction, involved in antioxidant response. These findings suggest constitutive protective mechanisms rather than extensive transcriptional reprogramming under PM exposure.

**Figure 6:**
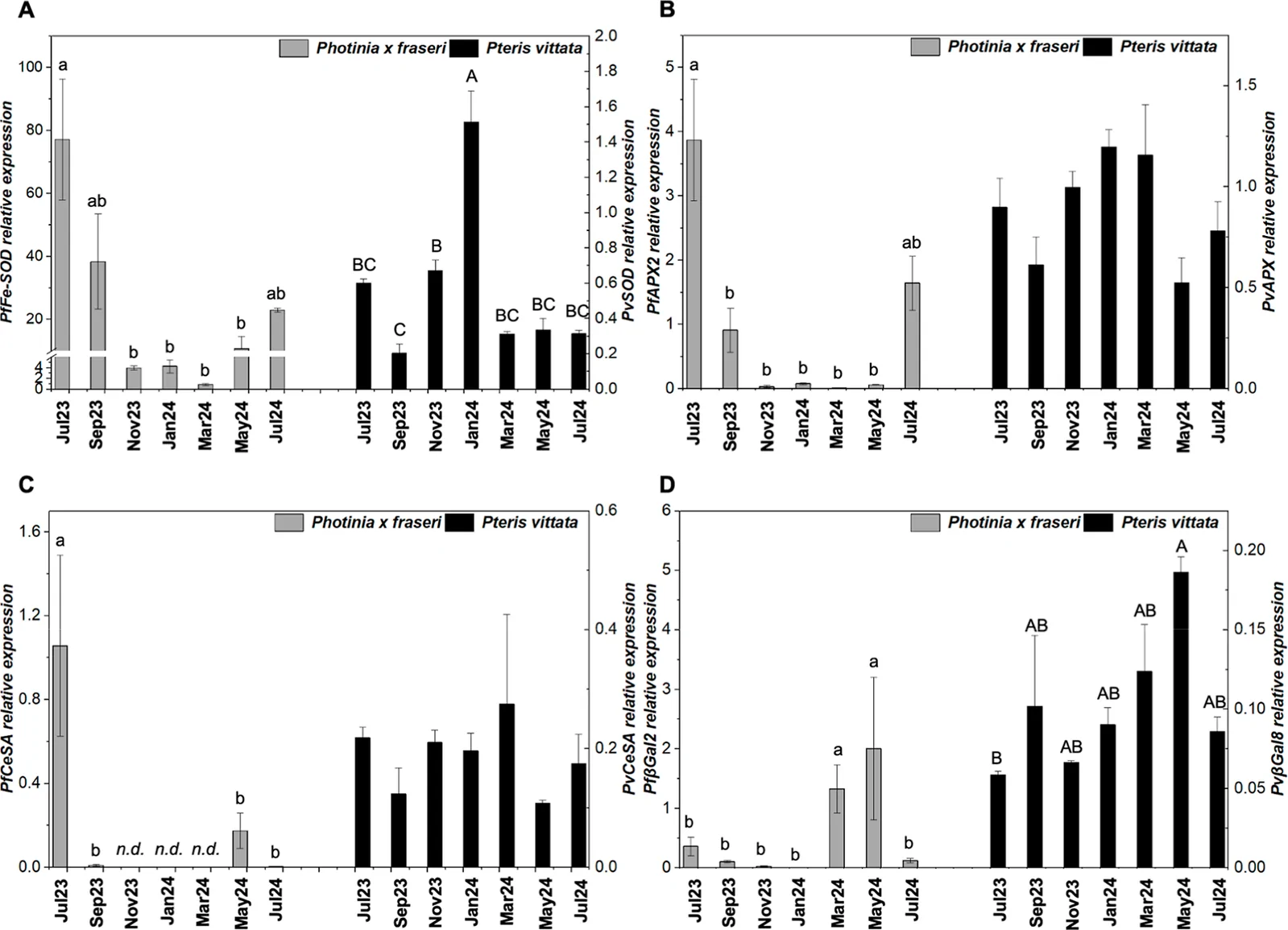
Relative expression of antioxidant defence-related and cell wall modification genes in *P. × fraseri* (A–D, left panel) and *P. vittata* (A–D, right panel) from July 2023 to July 2024. Gene expression was normalized to *PfTHF* in *P. × fraseri* and *PvEF1b* in *P. vittata*. Bars represent mean ± SE (*n* = 3). Different letters indicate significant differences among sampling times (one-way ANOVA, *p* ≤ 0.05), with lowercase letters referring to *P. × fraseri* and uppercase letters to *P. vittata*.

## 4 Conclusions

This one-year field study demonstrates that the reliability of plant-based PM biomonitoring depends not only on species-specific particle retention but also on the ability to distinguish externally deposited elements from endogenous elemental pools. Among the nine species investigated, *P. × fraseri* showed the highest and most temporally stable retention of traffic-related PM, whereas *P. vittata* exhibited the lowest and most variable retention. SEM–EDS analysis supported this contrasting behaviour, showing up to 2 orders of magnitude higher particle abundance on *P. × fraseri* leaves and substantial particle persistence after washing.

Chemical fractionation represented a key methodological contribution of the study. Traffic-related tracers were predominantly associated with the insoluble fraction, whereas soluble and leaf fractions were substantially influenced by endogenous elemental pools, including not only essential nutrients but also Cs and Rb. Thus, approaches based on total elemental concentrations alone may overestimate the contribution of atmospheric PM, while fractionation provides a more reliable basis for interpreting plant biomonitoring performance.

The integration of elemental, physiological, and molecular data further revealed contrasting species-specific responses under long-term urban exposure. The high and stable PM retention of *P. × fraseri* was associated with pronounced seasonal modulation of physiological traits and antioxidant- and cell wall-related gene expression, whereas *P. vittata* combined low PM retention with comparatively stable physiological and transcriptional profiles, consistent with more constitutive protective mechanisms. Overall, integrating chemical fractionation with microscopic, physiological, and gene expression analyses provides a transferable framework for evaluating both PM retention and plant responses and for supporting evidence-based species selection in urban green infrastructures.

Finally, long-term, very-low-volume PM10 sampling was conducted concurrently with the green wall biomonitoring campaign at several sites in its vicinity, including chemical characterization and oxidative potential analysis of PM10. The integration of these complementary datasets in a future study will allow the findings of the present work to be further explored by investigating the relationships between atmospheric PM composition, particles retained on leaf surfaces, and potential emission sources, while also providing insights into particle oxidative potential and its possible association with stress responses in the plant bioindicators. This integrated approach may ultimately strengthen the use of green walls as effective tools for biomonitoring atmospheric particulate pollution.

## Supplementary Material



## Notes

Additional experimental details, materials, and methods, including experimental setup figures and table (PDF)
