# Supplementary material for: Urban Green Walls under Traffic Stress: Linking the Elemental Composition of Particulate Matter Deposition to Plant Physiological and Gene Expression Responses
Source: Environ Sci Technol. 2026 Sep 18;60(38):27178–91. doi: 10.1021/acs.est.6c10086 (PMC13629620; doi:10.1021/acs.est.6c10086)
Supplement: Supplementary file 1 [file es6c10086_si_001.pdf]

Supplemental Material

Urban green walls under traffic stress: linking the elemental composition of particulate matter deposition to plant physiological and gene expression responses

Lorenzo Massimi[†1], Maria Luisa Antenozio[†2,3], Davide Marzi[2,3], Alice Zara[1], Caterina Tiraboschi[1,4], Federica Porcu[1,3], Laura Varone[1,3], Silvia Canepari[1], Tommaso Rossi[5], Adriana Pietrodangelo[5,3], Cinzia Perrino[5], Patrizia Brunetti[2,3*]

[1]Department of Environmental Biology, Sapienza University of Rome, P.le A. Moro 5, 00185 Rome, Italy;

[2]Research Institute on Terrestrial Ecosystems, National Research Council of Italy (CNR IRET), 00010, Rome, Italy;

[3]National Biodiversity Future Center (NBFC), 90133 Palermo, Italy;

[4]Department of Public Health and Infectious Diseases, Sapienza University of Rome, P.le A. Moro 5, 00185 Rome;

[5]Institute of Atmospheric Pollution Research National Research Council of Italy (CNR IIA), Via Salaria km 29.300, 00015 Monterotondo St, Rome, Italy

Table S1. Leaf Morphology traits of plant species used in this study.

Species	Family	Leaf type	Shape	Surface/Texture	Margins	Image	Reference
Pteris vittata	Pteridaceae	1-pinnate fronds	Linear-lanceolate pinnae	Glabrous, bright green	Finely serrated	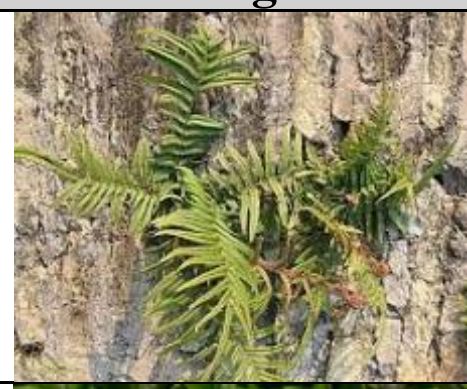	[1,2]
Viburnum tinus lucidum	Viburnaceae	Simple leaves	Ovate to elliptic	Glossy, leathery, dark green	Entire	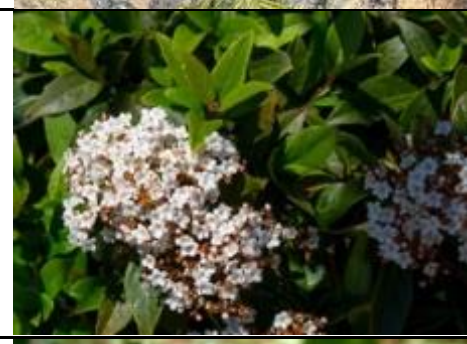	[1,3]
Buxus sempervirens	Buxaceae	Simple leaves	Elliptic	Glabrous, dark green, glossy above, matt and lighter below.	Curved	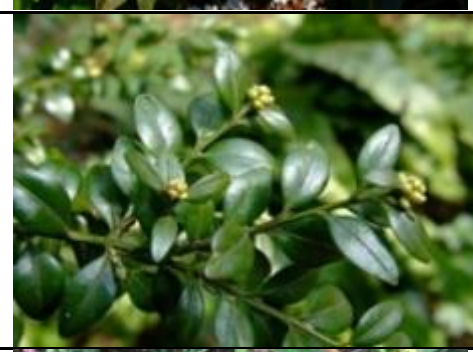	[3]
Nerium oleander	Apocynaceae	Simple leaves	Linear-lanceolate to narrowly elliptic	Glossy, leathery, dark green	Entire	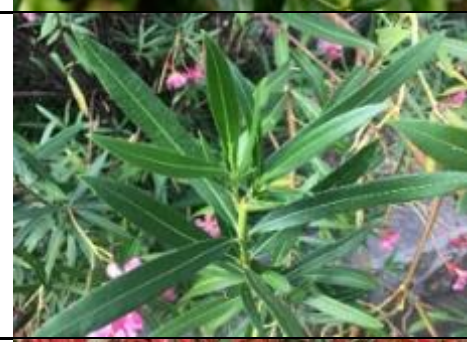	[1,3]
Photinia x fraseri	Rosaceae	Simple leaves	Ovate to elliptic	Glossy, dark green (older) or bright red (younger)	Dentate	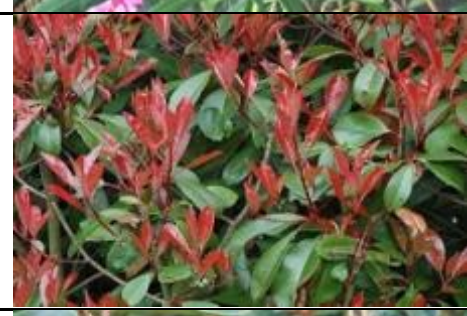	[3]
Elaeagnus ebbingei	Elaeagnaceae	Simple leaves	Elliptic	Dark green and glossy adaxially, silvery-grey abaxially	Entire	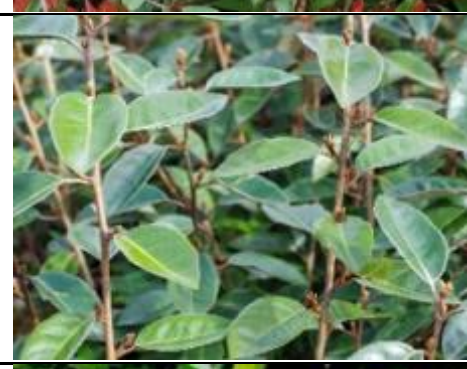	[3]
Prunus laurocerasus	*Rosacea*	Simple leaves	Elliptic to obovate	Glossy, leathery, glabrous	Serrulate or entire	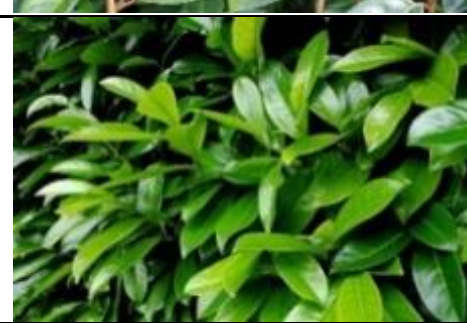	[1,3]
Geranium "Rozanne"	Geraniaceae	Palmately 5-angled leaves	Ovate-rhombic	Pilose-pubescent	Dentate	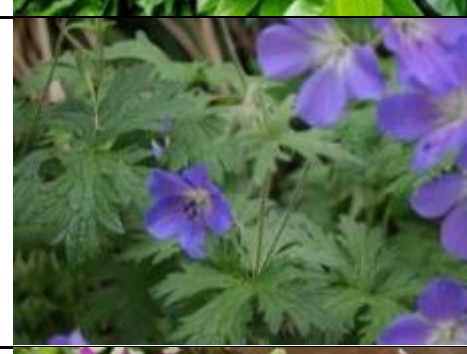	[1,4]
Drosanthemum hispidum	Aizoaceae	Simple leaves	Cylindrical	Glossy, densely papillate	Entire	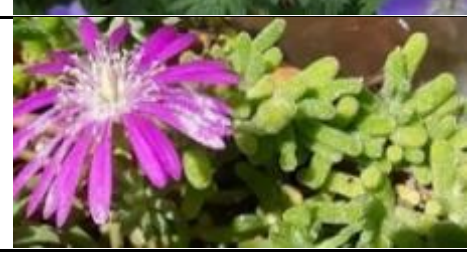	[1,5]

1.1 Experimental design and workflow (Figure S1)

<u>Phase 1 – Green wall installation and screening</u>

- Installation of a roadside green barrier along Via Nomentana
- 9 plant species monitored over 1 year (July 2023 – July 2024)
- 1-month acclimation period
- Monthly sampling (July–December 2023) of leaves
- Optimization of chemical fractionation protocol:
  Insoluble fraction (leaf surface-bound PM)
  Soluble fraction (loosely bound / wash-off fraction)
  Total leaf tissue fraction (internal elements/ residual elements from leaf washing)
- Analysis of the water-soluble, insoluble and leaf fraction of 27 elements

Objective: identify species-specific PM retention efficiency

Outcome: *Photinia × fraseri* identified as the most efficient species *Pteris vittata* identified as the least efficient species

<u>Phase 2 – Insoluble fractionation and SEM-EDS analyses in *P. × fraseri* and *P. vittata*</u>

- Focus on temporal trends of insoluble fraction of macro (Al, Fe) and micro (Cr, Mn, Ti, Zn) and traffic tracer elements (Cs, Mo, Rb, Sb, Sn)
- SEM–EDS characterization of PM retained on leaf surface.
- Relative contributions of PM deposition and plant physiological pool to elemental retention

Objectives: 1) Identify the species with the most stable retention of traffic-related tracers over time. 2) Discriminate between surface deposition and internal uptake of elements.

<u>Phase 3 – Physiological measurements and gene expression analysis in *P. × fraseri and P. vittata*</u>

Leaves sampling frequency: every 2 months. Period: July 2023 – July 2024

- *Physiological Parameters measured*: Fv/Fm → maximum PSII efficiency (photoinhibition indicator), Φy → actual photochemical efficiency, PRI → short-term photoprotective energy dissipation, ARI → anthocyanin content, NDVI → canopy greenness / photosynthetic activity, CRI1 → carotenoid-related photoprotection
- *Genes included in gene expression analyses*: antioxidant-related genes *(PvSOD, PvAPX* and *PfFe-SOD*, *PfAPX2*) and genes involved in cell wall biosynthesis and remodeling (*PvCesA, PvβGal8* and *PfCesA*, *PfβGal2*)

Objective: Evaluate plant physiological status and stress-response strategies.

<u>Averall Objective</u>: Link morphological and physiological traits as well as gene expression responses to PM exposure and elemental accumulation

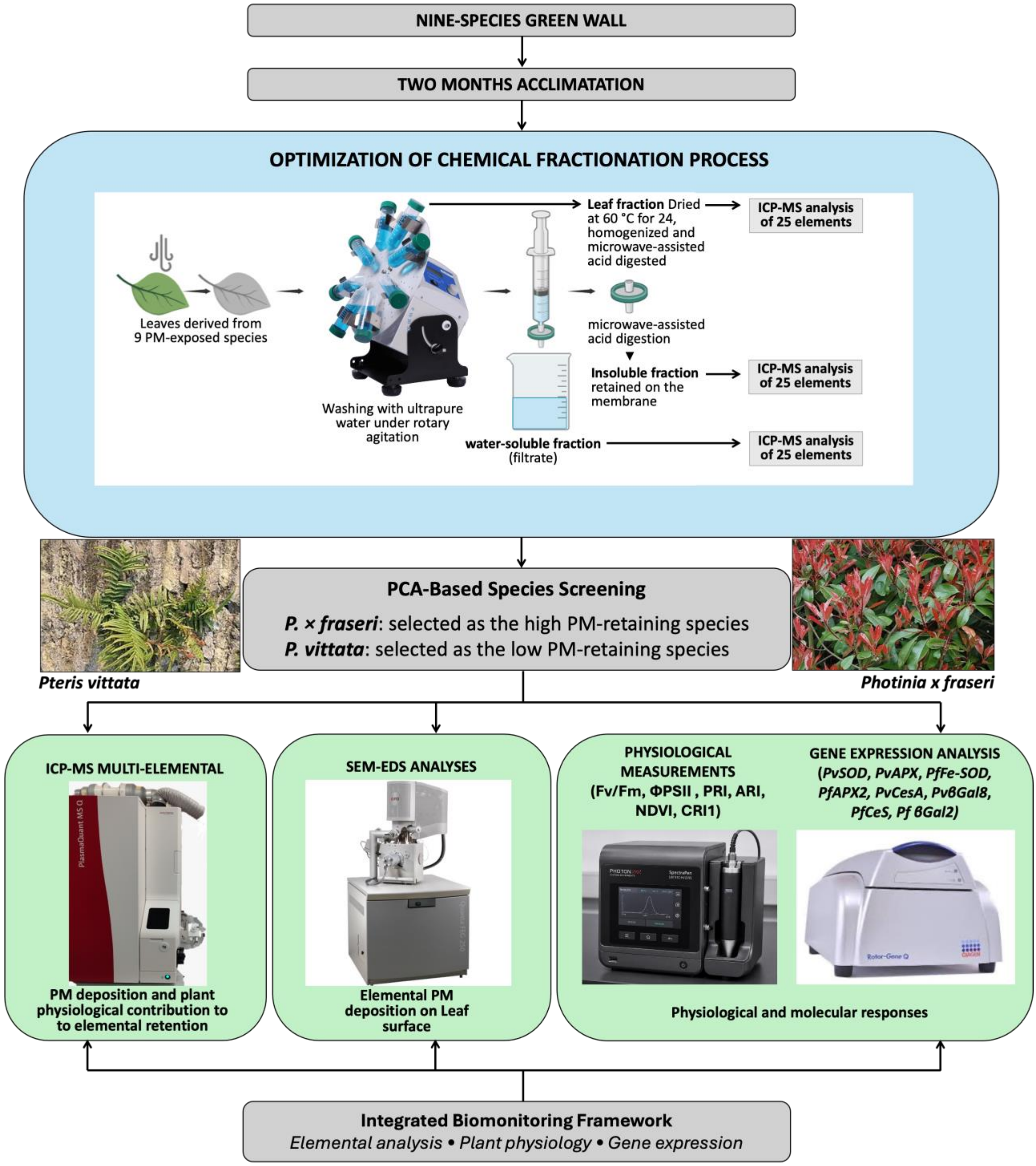

Figure S1. Schematic overview of the experimental workflow, including green wall installation, species screening based on PM retention, and subsequent detailed analyses (chemical fractionation for elemental analysis, physiological measurements, and gene expression) on selected species.

Table S2. Timeline of sampling activities performed throughout during the monitoring period. Following the installation of the green wall in May 2023, leaf sampling for chemical fractionation analyses was conducted monthly on all nine plant species from July to December 2023, whereas physiological and molecular analyses were performed every two months. From January to July 2024, all sampling and analyses (chemical, physiological, and molecular) were carried out every two months on the two selected species, *Pteris vittata* and *Photinia × fraseri*.

Sampling date	Chemical fractionation	Physiological analyses	Molecular analyses
July 2023	✓	✓	✓
August 2023	✓	–	–
September 2023	✓	✓	✓
October 2023	✓	–	–
November 2023	✓	✓	✓
December 2023	✓	–	–
January 2024	✓	✓	✓
March 2024	✓	✓	✓
May 2024	✓	✓	✓
July 2024	✓	✓	✓

1.2 Leaf morphometric parameters

Leaves were collected from *Pteris vittata* and *Photinia x fraseri* (n = 6 each) and scanned at high resolution. Digital images were analysed using imageJ software (ImageJ, IJ 1.46r, https://imagej.net/ij/). Leaf images were converted to binary format and analyzed using the "Analyze Particles" function to extract morphometric parameters, including leaf area (cm^2), perimeter (cm), circularity, aspect ratio (AR), major and minor axis lengths, roundness, and solidity. The major and minor axes correspond to those of the best-fitting ellipse describing the leaf outline. Images were calibrated using a known scale prior to analysis. For each parameters mean values ± standard error (SE) was calculated and used for subsequent comparisons **(Figure S2)**

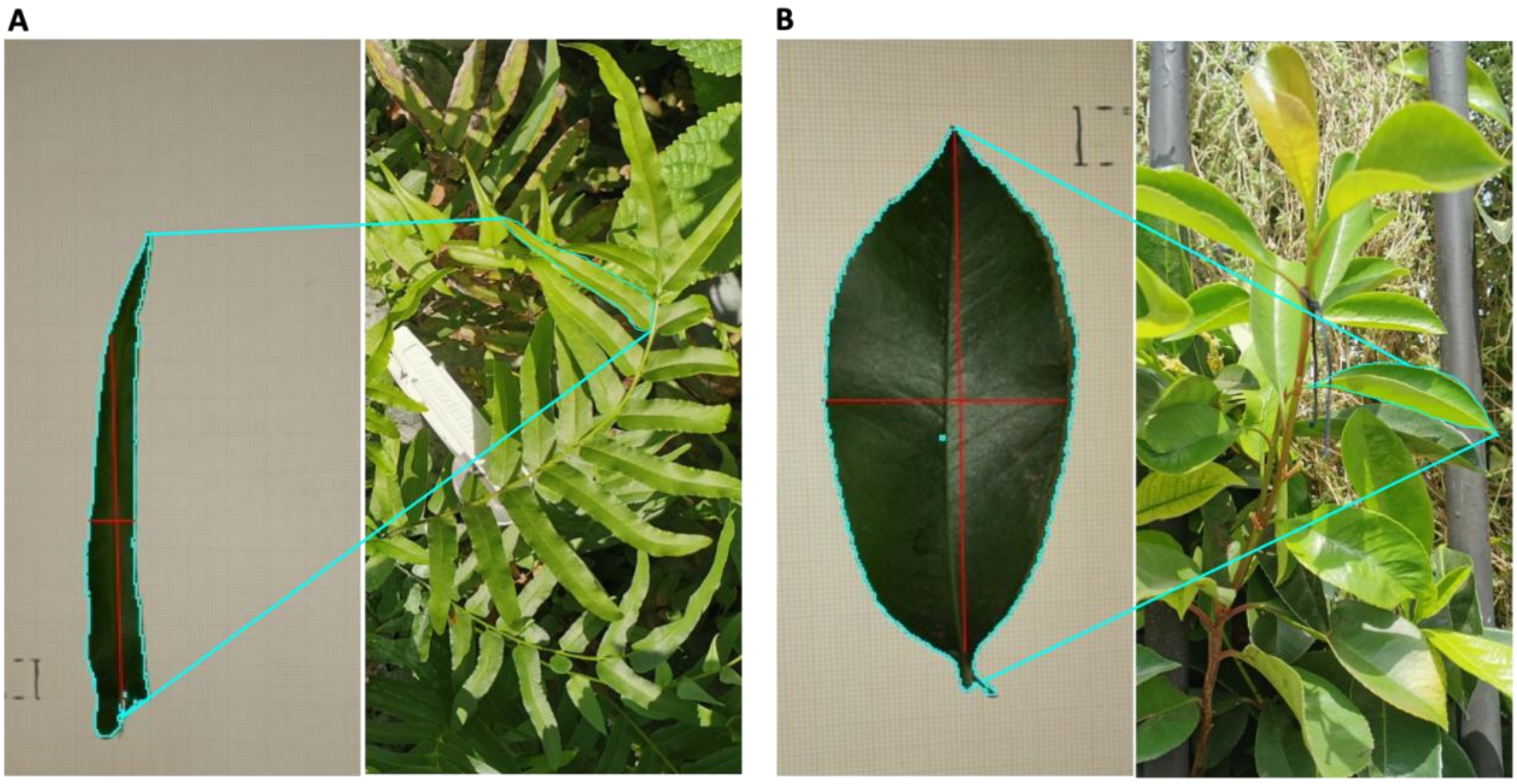

C

	Area (cm²)	Perimeter (cm)	Circularity	Aspect ratio (AR)	Major axis length (cm)	Minor axis length (cm)	Roundness	Solidity
Pteris vittata	7.252 ± 1.401	18.955 ± 3.019	0.268 ± 0.032	6.405 ± 0.825	7.675 ± 1.231	1.183 ± 0.036	0.164 ± 0.017	0.797 ± 0.045
Photinia x fraseri	31.114 ± 2.588	28.084 ± 1.417	0.497 ± 0.032	1.975 ± 0.079	8.793 ± 0.363	4.480 ± 0.252	0.510 ± 0.022	0.945 ± 0.002

Area = surface area of the leaf blade.
Perimeter = Length of the leaf outline.
Circularity = Dimensionless descriptor of shape compactness, calculated as 4π×Area/Perimeter^2; values closer to 1 indicate a more circular shape.
AR = Ratio between the major and minor axes of the fitted ellipse; higher values indicate more elongated shapes.
Major axis length = Length of the major axis of the ellipse fitted to the leaf outline, representing maximum leaf length.
Minor axis length = Length of the minor axis of the fitted ellipse, representing maximum leaf width perpendicular to the major axis.
Roundness = Dimensionless measure of shape compactness based on area and major axis length; lower values indicate more elongated leaves.
Solidity = Ratio between leaf area and the area of its convex hull; values close to 1 indicate smooth margins, whereas lower values indicate lobed or indented outlines.

Figure S2. Leaf morphometry of *Pteris vittata* and *Photinia × fraseri*. **(A–B)** Representative pinna of *Pteris vittata* **(A)** and leaf of *Photinia × fraseri* **(B)**, with major and minor axes (red) and leaf perimeter (light blue) overlaid. **(C)** Morphometric leaf parameters of both species (mean ± SE; n = 6).

Table S3. Concentrations of 25 elements in the leaf tissue fraction. Values are reported for all nine species from July to December 2023 and for *Pteris vittata* and *Photinia × fraseri* only from January to July 2024. Concentrations are expressed as ng cm^{-2}, except for Al, Fe, K, Mg, and Na, which are expressed as µg cm^{-2}.

Plant Name	Month	Al	As	Be	Bi	Cr	Cs	Fe	K	La	Li	Mg	Mn	Mo	Na	Ni	Pb	Rb	Sb	Sn	Ti	Tl	U	V	Zn	Zr
Pteris vittata	JUL-23	0,021	21	0,0036	0,026	0,33	0,037	0,12	61	0,13	0,31	3,3	63	3,1	0,32	0,33	0,086	6,2	0,019	< LOD	3,6	0,014	0,0042	0,05	46	0,15
Viburnum lucidum	JUL-23	1,7	7,6	0,42	0,75	11	5,2	2	700	3,3	5,3	44	470	2,8	6,3	19	3,1	300	2	3,2	110	0,52	0,16	6,5	420	5,5
Buxus sempervirens	JUL-23	0,21	6,6	0,028	0,29	7,4	1	0,39	990	0,32	1,4	39	84	21	4,9	7,2	1,5	350	0,27	< LOD	35	0,65	0,026	0,39	170	1,2
Nerium oleander	JUL-23	2,7	5,6	0,33	1,2	43	6,9	5,8	1100	3,9	14	69	2700	66	24	21	4,8	300	3,4	8,7	210	0,39	0,29	6,9	720	8,6
Photinia x fraseri	JUL-23	0,33	1,9	0,066	0,71	5,7	2,2	0,56	620	0,73	3,6	36	200	23	4,6	5	3,9	390	0,45	< LOD	51	0,78	0,04	0,79	170	1,7
Elaeagnus ebbingei	JUL-23	0,6	1,7	0,11	0,41	11	0,96	0,64	310	1,2	1,8	7	720	6,1	6,2	11	2,1	220	0,5	0,57	33	0,37	0,06	1,5	140	2
Prunus laurocerasus	JUL-23	0,32	3	0,043	0,19	4,3	0,35	0,46	550	0,61	4,9	22	180	2,2	1,9	3,1	0,65	100	0,27	< LOD	43	0,11	0,032	0,74	150	1,2
Geranium Rozanne	JUL-23	0,57	1,4	0,06	0,23	7,6	0,27	0,77	140	0,97	0,64	4,7	87	9,4	3,4	4,9	0,85	21	0,59	1,1	30	0,13	0,051	1,5	73	1,6
Drosanthemum hispidum	JUL-23	0,65	1,1	0,18	0,4	7,9	3,1	1	1200	1,4	11	91	890	43	110	7,5	1,6	160	0,7	0,22	75	0,31	0,076	1,5	590	2,4
Pteris vittata	AUG-23	0,059	20	0,012	0,074	0,83	0,048	0,14	67	0,19	0,13	4,8	34	3,2	0,44	0,81	0,28	6,9	0,064	< LOD	5,6	0,045	0,009	0,11	49	0,19
Viburnum lucidum	AUG-23	2,7	7,8	0,24	1,4	14	1,1	2,3	1200	3,6	2,5	58	1800	4,4	3,6	6,5	5,7	250	1,8	2,4	290	1	0,4	5,1	380	7
Buxus sempervirens	AUG-23	0,47	5,3	0,23	0,73	4,8	0,47	0,51	820	1,1	3,7	31	100	35	3,7	5,3	1,6	240	0,85	< LOD	60	0,27	0,74	1,4	210	1,5
Nerium oleander	AUG-23	3,2	4,1	0,84	1,4	25	4,3	5,8	780	5,3	30	72	7200	57	32	14	4,9	180	3,3	8,6	230	0,81	0,34	7,6	630	7,7
Photinia x fraseri	AUG-23	0,54	3	0,095	0,55	5,9	2,6	0,75	540	1,1	1,9	50	200	21	2,7	3,5	1,8	370	1	1	74	0,48	0,092	1,1	270	2,2
Elaeagnus ebbingei	AUG-23	0,64	2,4	0,079	0,53	5	1,1	0,79	470	1,5	1,3	19	620	46	1,4	5,3	1,5	160	0,64	1,3	63	0,26	0,081	2	240	3
Prunus laurocerasus	AUG-23	0,23	4,6	0,057	0,23	3,2	0,95	0,47	520	0,44	2,6	28	160	3,7	2,4	3,2	0,75	86	0,32	0,1	46	0,13	0,076	1,2	250	0,75
Geranium Rozanne	AUG-23	1,6	5,6	0,15	0,83	11	0,72	1,9	370	1,4	2,8	16	190	18	1,7	5,1	3,5	31	1,7	4,9	110	0,57	0,17	4,2	180	7,2
Drosanthemum hispidum	AUG-23	1,8	2	0,28	1,1	11	1,7	1,9	940	5	16	97	950	38	60	4,9	4,6	83	1,5	4,2	130	0,79	0,2	4,4	600	6,6
Pteris vittata	SEPT-23	0,13	15	0,021	0,096	1,2	0,052	0,2	62	0,24	0,14	4	38	3,7	0,37	0,57	0,24	6,7	0,13	0,055	8,7	0,036	0,015	0,27	65	0,27
Viburnum lucidum	SEPT-23	0,42	7,5	0,11	0,58	7,6	1,7	0,79	960	0,83	5,2	45	410	5,6	4,8	4,3	1,2	200	0,93	< LOD	100	0,18	0,12	2,3	450	1,7
Buxus sempervirens	SEPT-23	0,3	3,1	0,085	0,39	3,5	0,5	0,35	730	0,65	1,9	25	66	34	2,1	2,7	0,85	170	0,51	< LOD	45	0,18	0,077	0,69	130	1
Nerium oleander	SEPT-23	3,5	2,8	0,97	2,1	19	5,8	6,2	640	6,8	33	75	7300	63	21	10	5,8	180	3	5,3	370	0,87	0,39	8,7	710	8,7
Photinia x fraseri	SEPT-23	1,3	3,8	0,24	0,94	9,9	2,1	1,6	750	2,3	7,5	56	620	22	5,1	5,7	2,6	240	1,7	2,3	160	0,49	0,15	2,6	440	4,1
Elaeagnus ebbingei	SEPT-23	0,66	2,3	0,11	0,64	5,2	0,85	0,74	340	1,4	1,9	10	420	8	1,6	6	2,5	130	0,75	1,1	48	0,43	0,089	1,9	180	2,5
Prunus laurocerasus	SEPT-23	0,14	3,4	0,041	0,25	3	0,54	0,23	450	0,32	1,8	16	47	1,3	1,8	1,5	0,48	67	0,46	< LOD	29	0,07	0,099	0,4	140	0,45
Geranium Rozanne	SEPT-23	0,8	2,3	0,11	0,48	4,9	0,35	0,83	110	1,3	1,2	5,8	54	7,6	0,6	2,3	1,9	12	0,72	1,9	43	0,31	0,084	1,9	130	2,3
Drosanthemum hispidum	SEPT-23	0,36	0,94	0,1	0,42	3,4	0,46	0,77	980	0,59	14	130	570	55	49	4,3	1	73	0,51	< LOD	76	0,16	0,07	1,4	630	1,1
Pteris vittata	OCT-23	0,2	10	0,024	0,14	3,7	0,14	0,27	95	0,36	0,22	3,8	18	2	1,6	2,3	0,44	9,9	0,2	0,5	11	0,068	0,019	0,39	47	0,59
Viburnum lucidum	OCT-23	1,1	11	0,25	1,2	13	2,9	1,4	1000	2,2	3,3	51	400	10	6,1	7,7	3,8	290	1,7	2,2	120	0,59	0,2	3,4	480	3,9
Buxus sempervirens	OCT-23	0,22	4,6	0,082	0,55	5,1	0,39	0,27	790	0,36	2,5	26	45	17	3	4,4	1,6	170	0,5	< LOD	40	0,42	0,067	0,56	140	0,65
Nerium oleander	OCT-23	3,4	9	0,58	0,92	46	4,8	7,5	1200	4,3	23	100	5600	98	33	28	4,7	240	4,5	14	130	0,52	0,3	9,6	950	9,7
Photinia x fraseri	OCT-23	1,3	4,4	0,26	1,1	14	2,3	1,5	800	2,1	9,2	49	390	26	8,9	7,7	3,4	460	3,1	2,7	97	0,72	0,17	3	360	5,9
Elaeagnus ebbingei	OCT-23	1,1	2,2	0,2	0,73	9	1,3	1,3	430	2,1	1,9	14	430	4,4	2,2	3,8	2,7	150	1,3	2,7	68	0,46	0,13	2,8	180	3,9
Prunus laurocerasus	OCT-23	0,6	3,9	0,098	0,48	16	0,43	0,79	720	0,92	0,9	31	220	3,3	7,8	12	1,4	100	0,87	1,5	68	0,22	0,071	1,3	190	1,9
Geranium Rozanne	OCT-23	0,67	1	0,089	0,43	8,5	0,28	0,73	94	1,4	0,75	6,4	41	6,7	3,5	6,3	1,6	12	0,64	0,9	36	0,25	0,078	1,7	210	1,5
Drosanthemum hispidum	OCT-23	1,4	1,2	0,19	0,95	25	1	1,7	1100	2,1	14	120	350	27	84	18	3	96	1,9	1,8	110	0,48	0,15	3	720	4,2
Pteris vittata	NOV-23	0,26	20	0,017	0,097	3	0,1	0,56	92	0,32	0,26	4,1	52	2,6	1,5	0,77	0,35	20	0,47	1,7	11	0,0066	0,019	0,46	82	0,88
Viburnum lucidum	NOV-23	1,1	13	0,065	0,31	23	1,1	2,7	860	1,5	4,8	36	170	8,1	7	6,2	2	120	1,9	5,5	47	0,046	0,24	4,8	430	3,7
Buxus sempervirens	NOV-23	0,18	8,1	0,0058	0,047	7,4	0,5	0,68	640	5,9	4,6	27	43	39	2,9	3,9	0,71	190	0,53	1,1	12	0,035	0,053	1,4	220	0,89
Nerium oleander	NOV-23	3,4	12	0,31	0,91	56	6	8,4	1200	4	14	130	2900	130	18	34	4,6	280	4,8	15	130	0,17	0,25	11	1200	11
Photinia x fraseri	NOV-23	1,1	3,6	0,12	0,52	21	2,9	2,1	570	1,7	4,7	52	470	23	10	10	2,5	400	1,9	4,7	71	1,5	0,12	2,7	340	4,1
Elaeagnus ebbingei	NOV-23	1,7	4,2	0,18	0,39	17	1,2	2,6	330	2,4	2,9	24	180	4,7	2,4	8,4	3,3	140	2,1	6,6	70	0,49	0,14	3,5	330	5,6
Prunus laurocerasus	NOV-23	1,2	6,3	0,086	0,16	9,2	0,45	1,9	400	1,2	1,1	34	890	2,9	1,7	7,5	1,9	74	0,91	2,5	38	0,3	0,14	2,2	270	2,7
Geranium Rozanne	NOV-23	0,63	1,9	0,067	0,21	6	0,36	1,1	160	0,77	0,93	12	86	18	3,2	3	1,3	23	1,1	3,1	30	0,02	0,058	1,2	280	2,4
Drosanthemum hispidum	NOV-23	0,32	0,72	0,057	0,22	4,8	0,25	0,83	510	0,5	7,2	63	260	25	29	2,6	2,2	38	0,66	1,3	35	0,059	0,05	1,1	360	1,2
Pteris vittata	DEC-23	0,13	25	0,007	0,08	3,7	0,089	0,43	77	0,21	0,23	5,2	29	2,9	2,6	1,8	0,68	14	0,38	1,2	5,7	0,0093	0,013	0,47	110	0,54
Viburnum lucidum	DEC-23	2,1	11	0,28	0,43	27	4,2	3,2	750	2,7	6,5	52	350	8,2	28	19	4,2	170	3	9,4	72	0,097	0,21	5,4	610	6,2
Buxus sempervirens	DEC-23	0,21	5,2	0	0,095	19	0,41	1,1	550	0,36	2,6	35	83	33	13	13	0,73	120	0,64	1,4	14	0,042	0,054	2	170	1
Nerium oleander	DEC-23	3,7	5,9	0,26	1,3	73	5,2	8,2	1200	4	11	81	1700	90	28	36	6	230	5	15	140	0,19	0,26	10	1000	13
Photinia x fraseri	DEC-23	1,8	4,4	0,15	0,52	37	3,4	3,5	640	2,3	7,8	58	760	27	19	18	3,4	450	2,9	8,6	72	2,7	0,15	4,4	440	6,2
Elaeagnus ebbingei	DEC-23	1,3	2,2	0,12	0,56	17	0,68	2	300	1,6	1,4	18	150	13	6,8	13	3,2	140	1,8	5,8	54	0,26	0,11	2,2	340	4,6
Prunus laurocerasus	DEC-23	0,84	2,3	0,045	0,16	13	0,22	1,2	300	0,79	0,51	16	450	1,8	5,3	7,5	1,5	30	0,66	1,9	23	0,13	0,068	1,5	110	1,8
Geranium Rozanne	DEC-23	0,64	3,1	0,058	0,31	17	0,23	1,8	130	0,83	0,82	9,7	140	22	7	8,4	1,5	8	1,8	4,3	27	0,027	0,066	2,2	330	2,3
Drosanthemum hispidum	DEC-23	1,1	2,2	0,12	0,41	20	0,53	3,2	950	1,7	13	97	350	34	65	6,5	10	70	2,7	7,4	51	0,024	0,14	3,8	800	4,5
Pteris vittata	JAN-24	0,027	7,5	0,0016	0,06	2	0,041	0,14	60	0,071	0,057	1,5	7	0,82	1,5	1	0,1	6,8	0,13	0,46	2,4	0,021	0,0054	0,21	20	0,23
Photinia x fraseri	JAN-24	0,96	2,9	0,1	0,56	19	2,5	1,9	570	1,4	5,2	43	440	23	11	10	3,3	380	1,5	3,8	56	1,6	0,085	2,4	280	3,6
Pteris vittata	MAR-24	0,11	35	0,0079	0,08	3	0,094	0,41	100	0,25	0,4	6,4	69	4,5	2,2	1,6	0,57	16	0,3	0,82	7	0,018	0,013	0,39	120	0,52
Photinia x fraseri	MAR-24	1,6	4,8	0,16	0,92	32	4,2	3	950	2,3	8,5	70	720	38	17	17	5,5	630	2,5	6,1	92	2,6	0,14	3,9	450	5,9
Pteris vittata	MAY-24	0,1	42	0,0086	0,08	2,5	0,098	0,4	120	0,29	0,53	7,3	99	5,7	1,9	1,5	0,5	16	0,24	0,57	8	0,024	0,013	0,33	120	0,51
Photinia x fraseri	MAY-24	1,4	5	0,17	1,2	28	4,8	2,7	1200	2,3	9,1	80	680	46	17	16	7	760	2,2	4,3	110	2,5	0,14	3,5	470	5,7
Pteris vittata	JUL-24	0,061	28	0,008	0,06	1,2	0,061	0,22	84	0,2	0,32	5,2	65	4	0,88	0,87	0,29	9,8	0,11	0,15	5,7	0,028	0,0087	0,16	72	0,28
Photinia x fraseri	JUL-24	0,77	3,3	0,11	0,83	13	3,2	1,3	770	1,4	4,9	55	360	30	8	8,3	4,2	510	1,2	1,6	77	1,3	0,089	1,8	300	3,2

Table S4. Concentrations of 25 elements in the water soluble fraction. Values are reported for all nine species from July to December 2023 and for *Pteris vittata* and *Photinia × fraseri* only from January to July 2024. Concentrations are expressed as ng cm^{-2}, except for Al, Fe, K, Mg, and Na, which are expressed as µg cm^{-2}.

Plant Name	Month	Al	As	Be	Bi	Cr	Cs	Fe	K	La	Li	Mg	Mn	Mo	Na	Ni	Pb	Rb	Sb	Sn	Ti	Tl	U	V	Zn	Zr
Pteris vittata	JUL-23	0,0041	59	0,0016	0,018	0,66	0,12	< LOD	81	0,018	3,3	3,5	27	0,95	0,66	0,64	0,075	25	0,045	0,067	0,2	0,1	0,011	0,17	61	0,048
Viburnum lucidum	JUL-23	0,005	5,5	0,0047	0,052	1,3	0,3	0,013	17	0,01	0,42	3,9	15	1,9	0,64	1,1	0,18	28	0,34	0,062	0,22	0,22	0,011	0,24	53	0,049
Buxus sempervirens	JUL-23	0,0059	0,14	0,0012	0,00067	0,54	0,062	0,015	14	0,02	0,088	0,91	3,3	0,72	0,47	0,51	0,014	22	0,031	0,0077	0,37	0,12	0,0027	0,14	130	0,022
Nerium oleander	JUL-23	0,031	0,097	0,023	0,077	0,92	0,28	0,033	12	0,099	0,57	1,3	35	0,46	2	1,4	0,55	18	0,18	0,18	0,55	0,23	0,023	0,31	390	0,17
Photinia x fraseri	JUL-23	0,028	0,19	0,015	0,04	1,2	0,27	0,034	24	0,057	0,3	1,7	12	0,59	0,55	0,51	0,14	65	0,088	0,052	0,59	0,23	0,0098	0,44	73	0,16
Elaeagnus ebbingei	JUL-23	0,009	0,15	0,0071	0,012	0,51	0,029	0,019	3,1	0,032	0,21	0,7	50	0,48	1,5	0,42	0,052	8,3	0,06	0,038	0,39	0,084	0,0078	0,23	78	0,059
Prunus laurocerasus	JUL-23	0,009	0,27	0,0048	0,0099	0,55	0,037	0,016	12	0,024	0,22	0,73	8,8	0,19	0,46	0,55	0,043	11	0,053	0,027	0,22	0,061	0,0037	0,26	69	0,049
Geranium Rozanne	JUL-23	0,0034	0,15	0,015	0,012	1,7	0,11	0,022	59	0,075	0,22	0,85	15	0,08	0,65	0,45	0,049	23	0,085	0,0077	0,19	0,065	0,0034	0,45	130	0,019
Drosanthemum hispidum	JUL-23	0,056	0,35	0,037	0,073	2,6	2,7	0,061	400	0,042	4,4	30	210	9	160	1,9	0,3	140	0,16	0,094	0,88	0,61	0,014	0,64	190	0,21
Pteris vittata	AUG-23	0,034	120	0,0048	0,11	2,3	0,2	< LOD	180	0,064	0,42	4,7	13	1,9	1,2	5,8	0,48	56	0,11	0,13	0,65	0,61	0,012	0,59	160	0,12
Viburnum lucidum	AUG-23	0,02	0,36	0,014	0,062	1,1	0,095	0,018	63	0,062	0,13	9,1	140	0,3	0,34	0,85	0,2	39	0,11	0,096	0,2	0,31	0,035	0,26	55	0,13
Buxus sempervirens	AUG-23	0,0066	0,27	0,014	0,0067	0,46	0,049	0,014	16	0,031	0,28	1,7	9,9	1,3	0,55	0,87	0,029	23	0,062	0,029	0,42	0,044	0,026	0,096	140	0,042
Nerium oleander	AUG-23	0,028	0,11	0,027	0,047	1,4	0,11	0,06	14	0,15	0,89	2,1	97	0,61	2,6	6,5	3,9	13	0,18	0,81	0,67	0,015	0,033	0,49	520	0,16
Photinia x fraseri	AUG-23	0,016	0,17	0,017	0,039	1,3	0,11	0,032	9,1	0,039	0,12	1,4	16	0,39	0,74	0,57	0,12	26	0,16	0,1	0,57	0,17	0,0091	0,47	85	0,16
Elaeagnus ebbingei	AUG-23	0,012	0,051	0,011	0,016	0,53	0,046	0,015	5,8	0,055	0,072	0,88	29	2,1	0,62	0,47	0,064	9,8	0,053	0,04	0,31	0,086	0,0092	0,18	91	0,11
Prunus laurocerasus	AUG-23	0,006	0,34	0,0053	0,023	0,6	0,24	0,013	52	0,057	0,64	6,1	17	0,55	0,15	0,48	0,087	25	0,027	0,02	0,2	0,11	0,0037	0,17	86	0,044
Geranium Rozanne	AUG-23	0,0043	0,32	0,017	0,038	2,1	0,11	0,022	82	0,088	0,58	1,5	14	0,2	0,28	0,56	0,15	17	0,077	0,018	0,23	0,19	0,0038	0,52	100	0,084
Drosanthemum hispidum	AUG-23	0,046	0,5	0,021	0,055	2,7	1,5	0,069	430	0,051	8,1	39	220	13	110	1,9	0,2	100	0,26	0,1	0,97	0,45	0,0075	0,65	210	0,28
Pteris vittata	SEPT-23	0,02	82	0,013	0,22	2	0,28	< LOD	150	0,12	0,45	4,4	18	1,1	0,94	2,6	0,76	69	0,083	0,1	0,53	0,024	0,016	0,52	92	0,45
Viburnum lucidum	SEPT-23	0,016	0,14	0,01	0,03	1,6	0,3	0,034	41	0,12	0,67	4,6	50	0,45	0,86	1,6	0,46	39	0,019	0,076	0,29	0,025	0,0076	0,44	90	0,087
Buxus sempervirens	SEPT-23	0,068	< LOD	0,011	0,02	0,46	0,019	0,016	5,8	0,14	0,12	0,51	8	0,45	1,5	3,5	3,3	6,4	0,15	0,44	0,56	0,01	0,015	0,064	170	0,088
Nerium oleander	SEPT-23	0,035	0,12	0,028	0,042	1,6	0,1	0,059	12	0,25	1,4	1,9	130	0,59	3,1	12	7,7	13	0,17	1,5	0,88	0,014	0,028	0,5	940	0,17
Photinia x fraseri	SEPT-23	0,012	0,17	0,019	0,033	1,5	0,16	0,031	26	0,054	0,87	2,8	35	0,76	1,2	3,4	1,2	33	0,22	0,2	0,54	0,068	0,011	0,51	99	0,13
Elaeagnus ebbingei	SEPT-23	0,016	0,14	0,011	0,0097	0,45	0,035	0,0084	7,4	0,097	0,088	0,65	46	1,2	1,6	1,3	0,59	9,1	0,069	0,13	0,2	0,02	0,0093	0,11	85	0,086
Prunus laurocerasus	SEPT-23	0,015	0,063	0,0066	0,018	0,89	0,023	0,014	5,6	0,13	0,071	0,42	6,1	0,12	0,73	3,2	1,5	4	0,046	0,37	0,24	0,0039	0,0073	0,1	140	0,051
Geranium Rozanne	SEPT-23	0,006	0,22	0,016	0,0034	1,8	0,11	0,016	48	0,098	0,66	1,4	17	0,14	0,37	1,1	0,4	17	0,07	0,011	0,2	0,017	0,0043	0,44	53	0,037
Drosanthemum hispidum	SEPT-23	0,17	1	0,0075	0,029	2,5	0,42	0,067	290	0,1	5,6	39	150	12	67	3,6	0,56	97	0,075	0,1	0,94	0,049	0,006	0,59	200	0,51
Pteris vittata	OCT-23	0,0082	26	0,0056	0,045	0,44	0,1	< LOD	36	0,052	0,057	0,73	3,6	0,27	0,27	0,43	0,39	15	0,042	0,045	0,17	0,0077	0,0063	0,12	73	0,081
Viburnum lucidum	OCT-23	0,017	0,061	0,014	0,033	0,51	0,084	0,002	14	0,055	0,1	1,4	13	0,33	0,36	0,69	0,35	15	0,026	0,057	0,27	0,017	0,0066	0,19	50	0,088
Buxus sempervirens	OCT-23	0,013	0,096	0,0095	0,024	1,1	0,052	0,02	24	0,1	0,28	1,4	9,1	1,2	1,2	2,4	1,2	24	0,11	0,32	0,34	0,12	0,014	0,27	150	0,11
Nerium oleander	OCT-23	0,067	0,033	0,025	0,056	1,3	0,11	0,056	14	0,15	0,47	2,1	50	0,61	2,1	3,8	4,7	11	0,18	0,59	0,53	0,013	0,03	0,44	310	0,17
Photinia x fraseri	OCT-23	0,011	0,15	0,021	0,024	0,77	0,057	0,037	14	0,045	0,36	1,1	26	0,34	1,2	1,1	0,43	25	0,25	0,15	0,64	0,032	0,012	0,38	52	0,16
Elaeagnus ebbingei	OCT-23	0,0099	0,071	0,013	0,0084	0,45	0,023	0,022	4,8	0,044	0,11	0,75	34	0,61	1,9	0,74	0,33	5,5	0,12	0,079	0,41	0,011	0,01	0,25	38	0,1
Prunus laurocerasus	OCT-23	0,015	0,015	0,015	0,013	1,9	0,047	0,031	33	0,15	0,039	4	32	0,21	0,5	1	0,49	22	0,1	0,11	0,39	0,019	0,013	0,62	55	0,1
Geranium Rozanne	OCT-23	0,0068	0,24	0,017	0,0069	1,7	0,075	0,021	31	0,098	0,24	1,7	15	0,15	0,2	0,54	0,56	14	0,072	0,0087	0,13	0,0086	0,0081	0,33	160	0,041
Drosanthemum hispidum	OCT-23	0,023	0,37	0,006	0,011	2	0,46	0,033	220	0,035	3	22	56	4	52	1	0,4	73	0,08	0,073	0,5	0,041	0,0045	0,46	110	0,21
Pteris vittata	NOV-23	0,0027	11	0,002	0,014	0,18	0,028	< LOD	23	0,029	0,022	0,38	3,8	0,064	0,55	0,39	0,077	10	0,023	0,023	0,088	0,0036	0,0029	0,054	31	0,016
Viburnum lucidum	NOV-23	0,013	0,1	0,0028	0,015	1,1	0,067	0,018	20	0,072	0,25	2,1	7,4	0,24	0,5	1,2	0,27	9,6	0,012	0,049	0,058	0,0084	0,0039	0,32	45	0,03
Buxus sempervirens	NOV-23	0,014	0,022	0,0041	0,019	0,52	0,028	0,0082	15	0,072	0,19	0,69	4,1	0,67	0,55	1,5	0,89	12	0,05	0,2	0,13	0,0081	0,0065	0,12	92	0,074
Nerium oleander	NOV-23	0,091	< LOD	0,015	0,043	1	0,054	0,032	8,5	0,21	0,32	1,4	14	0,4	1,3	5,1	7,3	5,8	0,11	0,82	0,42	0,0065	0,012	0,26	380	0,13
Photinia x fraseri	NOV-23	0,014	0,14	0,019	0,04	1,1	0,11	0,036	12	0,035	0,22	1,5	15	0,38	1,7	0,82	0,17	28	0,22	0,14	0,54	0,12	0,0099	0,43	73	0,15
Elaeagnus ebbingei	NOV-23	0,0036	< LOD	0,0039	0,0098	0,11	0,01	0,009	3	0,022	0,058	0,57	3,8	0,3	1,6	0,28	0,22	2	0,058	0,047	0,098	0,008	0,0021	0,1	28	0,041
Prunus laurocerasus	NOV-23	0,0086	0,058	0,0074	0,014	0,91	0,013	0,031	5,9	0,033	0,098	0,83	25	0,17	2	1,5	0,16	4,3	0,22	0,11	0,44	0,0055	0,029	0,36	68	0,15
Geranium Rozanne	NOV-23	0,0039	0,17	0,01	0,0059	1	0,16	0,013	42	0,094	0,51	2,5	9,3	0,24	6,6	0,39	0,46	21	0,07	0,0086	0,14	0,0082	0,0031	0,24	61	0,027
Drosanthemum hispidum	NOV-23	0,12	1	0,011	0,036	3,5	0,41	0,085	350	0,18	12	50	150	14	76	3,4	0,92	110	0,2	0,21	1,1	0,064	0,0085	0,88	290	0,54
Pteris vittata	DEC-23	0,0057	16	0,0038	0,031	0,35	0,056	< LOD	28	0,06	0,096	0,57	2,4	0,11	1,1	0,95	0,22	14	0,087	0,09	0,15	0,0059	0,0047	0,087	70	0,044
Viburnum lucidum	DEC-23	0,012	1,2	0,011	0,039	1,3	0,24	0,03	18	0,039	3,2	3	30	0,47	1,3	1,6	0,23	18	0,075	0,063	0,33	0,0067	0,01	0,45	77	0,089
Buxus sempervirens	DEC-23	0,014	0,081	0,0067	0,024	0,56	0,031	0,0086	14	0,1	0,18	0,89	4,6	0,69	1,5	2,3	0,67	9,8	0,12	0,18	0,2	0,0067	0,012	0,14	120	0,071
Nerium oleander	DEC-23	0,016	< LOD	0,016	0,031	0,47	0,027	0,019	4,7	0,11	0,15	1,2	13	0,29	2,1	1,9	0,71	3,4	0,18	0,2	0,37	0,004	0,0092	0,22	130	0,12
Photinia x fraseri	DEC-23	0,0085	0,085	0,018	0,033	0,73	0,095	0,033	13	0,025	0,29	1,3	11	0,3	2,4	0,92	0,19	25	0,24	0,16	0,42	0,049	0,0089	0,31	47	0,11
Elaeagnus ebbingei	DEC-23	0,007	0,02	0,005	0,0097	0,3	0,022	0,015	5,4	0,032	0,13	0,89	8,9	0,35	2,8	0,63	0,18	5,2	0,085	0,054	0,18	0,01	0,0042	0,14	43	0,041
Prunus laurocerasus	DEC-23	0,007	0,014	0,01	0,017	1,2	0,015	0,025	14	0,064	0,036	1,6	43	0,1	0,9	1,1	0,22	5,8	0,033	0,052	0,21	0,0097	0,019	0,37	59	0,066
Geranium Rozanne	DEC-23	0,0038	0,47	0,01	0,0058	1,9	0,14	0,056	45	0,046	1,1	5,2	29	0,35	2,9	1,1	0,34	18	0,11	0,012	0,24	0,016	0,0036	0,51	85	0,025
Drosanthemum hispidum	DEC-23	0,023	0,69	0,012	0,033	3,7	0,29	0,082	310	0,21	17	48	96	12	64	2,2	1,1	91	0,28	0,28	0,94	0,062	0,0089	0,96	300	0,43
Pteris vittata	JAN-24	0,0027	10	0,0021	0,017	0,18	0,044	< LOD	21	0,026	0,065	0,24	1,5	0,088	0,4	0,2	0,057	11	0,041	0,03	0,085	0,0037	0,0027	0,062	18	0,037
Photinia x fraseri	JAN-24	0,017	0,12	0,015	0,034	0,89	0,17	0,03	17	0,038	0,27	1,4	10	0,41	1,3	0,65	0,15	42	0,15	0,094	0,46	0,13	0,0085	0,34	55	0,12
Pteris vittata	MAR-24	0,0074	56	0,004	0,037	0,76	0,13	< LOD	81	0,059	2,6	3	22	0,79	1,3	1,2	0,22	29	0,099	0,12	0,26	0,083	0,012	0,19	98	0,069
Photinia x fraseri	MAR-24	0,027	0,2	0,024	0,055	1,5	0,28	0,05	28	0,061	0,44	2,2	17	0,67	2,2	1,1	0,25	67	0,24	0,16	0,76	0,21	0,014	0,56	90	0,2
Pteris vittata	MAY-24	0,0087	86	0,0042	0,042	1,1	0,18	< LOD	120	0,057	4,4	4,9	37	1,3	1,5	1,4	0,22	41	0,11	0,14	0,35	0,14	0,017	0,27	120	0,088
Photinia x fraseri	MAY-24	0,041	0,29	0,03	0,071	2	0,41	0,063	39	0,088	0,55	2,9	22	0,94	2,1	1,2	0,29	100	0,25	0,16	1	0,33	0,018	0,74	120	0,27
Pteris vittata	JUL-24	0,015	88	0,0035	0,056	1,3	0,17	< LOD	130	0,046	2,7	4,3	26	1,4	1,1	2,6	0,26	41	0,089	0,11	0,4	0,28	0,013	0,34	110	0,085
Photinia x fraseri	JUL-24	0,028	0,22	0,02	0,05	1,5	0,27	0,043	24	0,061	0,32	2	17	0,64	1,1	0,76	0,19	64	0,17	0,1	0,72	0,24	0,012	0,55	94	0,2

Table S5. Concentrations of 25 elements in the insoluble fraction. Values are reported for all nine species from July to December 2023 and for *Pteris vittata* and *Photinia × fraseri* only from January to July 2024. Concentrations are expressed as ng cm^{-2}, except for Al, Fe, K, Mg, and Na, which are expressed as µg cm^{-2}.

Plant Name	Month	Al	As	Be	Bi	Cr	Cs	Fe	K	La	Li	Mg	Mn	Mo	Na	Ni	Pb	Rb	Sb	Sn	Ti	Tl	U	V	Zn	Zr
Pteris vittata	JUL-23	2,9	3,7	0,22	1,3	90	1	3,3	37	3,8	2,1	1,3	56	2,3	30	33	5,7	11	4,5	13	110	0,89	0,2	5,6	210	9,1
Viburnum lucidum	JUL-23	2,3	0,83	0,2	1	51	0,87	2,5	10	3,5	1,9	0,86	38	1,4	17	24	4,8	8,9	2,2	7,7	93	0,77	0,2	5,2	180	7,3
Buxus sempervirens	JUL-23	10	3,5	0,73	3,7	150	2,7	10	28	13	7,1	2,7	130	5,8	33	44	15	29	9,5	31	380	2,3	0,67	20	340	27
Nerium oleander	JUL-23	13	4,1	1,1	4,3	200	4,8	12	32	20	9,2	3,9	190	8	46	74	17	43	12	35	580	2,7	0,98	27	260	41
Photinia x fraseri	JUL-23	24	8	2	9,5	190	8,7	22	48	39	16	5,4	330	13	24	49	43	90	15	64	920	6,9	2	52	560	66
Elaeagnus ebbingei	JUL-23	10	3,4	0,91	6,5	100	3,9	8,7	22	16	6,9	2,5	160	4	22	27	24	37	6,8	21	420	3,5	0,93	22	280	30
Prunus laurocerasus	JUL-23	22	7	1,9	7,4	150	7,8	20	36	34	15	4,4	310	10	19	35	32	73	12	49	820	5	2	47	360	58
Geranium Rozanne	JUL-23	7,6	2,2	0,63	3,3	72	2,8	7,2	36	12	4,9	2,2	100	5,3	15	20	14	28	6,7	22	310	2,2	0,67	17	190	26
Drosanthemum hispidum	JUL-23	6,9	2,9	0,58	3,6	260	2,6	8,3	110	12	5,3	5,5	160	7,3	81	76	12	31	12	36	300	1,8	0,57	14	410	26
Pteris vittata	AUG-23	9,9	11	0,73	4,4	270	3,5	11	110	13	7,3	4,2	180	7,3	96	100	19	37	14	39	390	3,1	0,66	19	750	30
Viburnum lucidum	AUG-23	2,6	0,93	0,22	1,1	53	0,97	2,7	11	4	2	0,93	42	1,6	17	26	5,3	9,9	2,4	8,6	100	0,86	0,22	5,8	190	8,1
Buxus sempervirens	AUG-23	10	3,7	0,77	3,8	150	2,9	10	28	14	7,3	2,8	140	5,7	33	45	16	31	9,3	30	410	2,4	0,7	20	360	28
Nerium oleander	AUG-23	14	4,3	1,2	4,6	210	5	13	34	21	9,7	4,1	200	8,6	49	79	19	45	13	37	610	2,9	1	29	270	44
Photinia x fraseri	AUG-23	28	9,2	2,3	11	210	10	26	54	45	19	6,1	380	14	24	54	49	110	16	69	1100	7,9	2,4	60	650	75
Elaeagnus ebbingei	AUG-23	12	3,9	1,1	7,7	100	4,5	9,9	24	19	8	2,8	180	4,3	21	27	28	43	7,4	23	480	4,1	1,1	26	310	34
Prunus laurocerasus	AUG-23	17	5,5	1,5	5,9	120	6,2	16	30	27	12	3,6	250	8	17	30	25	59	9,9	39	650	4	1,6	37	300	47
Geranium Rozanne	AUG-23	8,7	2,5	0,72	3,6	76	3,1	7,8	39	14	5,6	2,4	110	5,4	16	22	16	32	6,7	22	350	2,5	0,75	19	210	28
Drosanthemum hispidum	AUG-23	5,6	2,4	0,45	2,8	230	2,2	6,6	110	9,2	4,4	5	140	6	80	73	9,9	26	9,5	28	230	1,5	0,46	11	350	20
Pteris vittata	SEPT-23	8,7	8,4	0,63	3,7	200	3,1	8,8	77	11	6,5	3,5	140	5,8	76	81	17	30	10	29	350	2,8	0,55	16	670	25
Viburnum lucidum	SEPT-23	4,8	1,8	0,42	2,1	90	1,8	5,1	19	7,5	3,5	1,7	77	3	30	47	9,9	18	4,5	16	200	1,6	0,42	11	350	15
Buxus sempervirens	SEPT-23	13	4,7	0,97	4,6	180	3,6	12	34	17	9	3,4	170	6,5	40	56	20	39	11	34	510	3,1	0,89	25	450	35
Nerium oleander	SEPT-23	17	5,1	1,4	4,9	220	5,9	15	37	24	11	4,6	230	8,6	52	86	21	52	14	39	730	3,4	1,2	33	310	50
Photinia x fraseri	SEPT-23	34	11	2,9	12	240	13	31	63	56	23	7,2	450	17	24	60	58	130	18	77	1300	9,3	2,9	73	790	89
Elaeagnus ebbingei	SEPT-23	4,9	1,5	0,4	1,9	60	1,8	4,2	31	7,6	3,3	1,7	64	2,8	17	19	8,8	18	3,5	12	190	1,4	0,4	10	140	15
Prunus laurocerasus	SEPT-23	15	4,5	1,2	4,9	110	5,1	13	26	22	9,9	3,1	210	6,7	17	28	21	48	8,4	33	550	3,3	1,3	31	270	39
Geranium Rozanne	SEPT-23	7,3	2,2	0,61	2,8	58	2,6	6,2	30	11	4,8	1,9	85	3,7	13	17	13	26	4,6	16	290	2,2	0,61	16	170	22
Drosanthemum hispidum	SEPT-23	3,4	1,6	0,25	1,6	180	1,4	3,9	94	5,3	3	3,9	94	3,9	70	62	5,9	18	5,1	15	140	0,89	0,27	6,6	250	11
Pteris vittata	OCT-23	3,1	2,3	0,22	1,2	57	1,1	2,8	20	3,8	2,3	1,1	41	1,7	23	24	6,1	9,6	2,7	7,8	120	0,99	0,18	5,5	240	8,3
Viburnum lucidum	OCT-23	5,8	2,1	0,51	2,4	99	2,2	6,1	21	9	4	1,9	90	3,5	33	54	12	22	5,4	19	230	1,9	0,5	13	410	18
Buxus sempervirens	OCT-23	15	5,4	1,1	5,1	200	4,2	13	37	19	10	3,9	190	6,5	42	63	23	45	11	35	600	3,6	1	29	520	39
Nerium oleander	OCT-23	13	3,8	1,1	5,1	240	4,7	13	36	21	9	4,1	210	10	55	86	18	45	14	44	560	2,7	1,1	29	260	43
Photinia x fraseri	OCT-23	36	12	3,1	13	240	14	33	66	61	25	7,4	470	17	20	60	60	140	17	75	1400	9,7	3,1	78	840	94
Elaeagnus ebbingei	OCT-23	4,9	1,5	0,4	1,7	54	1,8	3,9	30	7,5	3,4	1,6	59	2,1	18	19	8,9	18	2,3	8,6	190	1,5	0,4	10	130	14
Prunus laurocerasus	OCT-23	22	6,6	1,8	7,8	150	7,9	17	78	34	15	5,2	230	8,5	35	48	40	76	10	38	850	6,7	1,8	46	500	63
Geranium Rozanne	OCT-23	7,6	2,3	0,63	2,7	52	2,7	5,9	27	12	5,1	1,8	81	2,9	12	16	14	26	3,4	13	290	2,3	0,62	16	170	22
Drosanthemum hispidum	OCT-23	1,6	0,9	0,076	0,6	150	0,87	1,5	99	1,8	2	3,4	65	2,4	74	63	2,6	12	1,2	4,4	54	0,41	0,11	2,5	180	3,9
Pteris vittata	NOV-23	0,9	1,7	0,074	0,48	42	0,31	1,3	18	1,3	0,64	0,48	24	0,99	13	14	1,7	4,5	2,2	6,1	36	0,26	0,073	1,9	63	3,4
Viburnum lucidum	NOV-23	0,51	0,14	0,034	0,26	29	0,2	0,63	5	0,67	0,84	0,33	12	0,37	8,6	8,5	1,1	2,1	0,57	1,9	20	0,17	0,037	1	59	1,6
Buxus sempervirens	NOV-23	8,4	2,8	0,59	3,2	140	2,2	9,3	25	11	6,2	2,3	110	6,1	32	38	12	23	10	32	300	1,8	0,52	16	270	23
Nerium oleander	NOV-23	10	3	0,89	4,3	200	3,7	11	30	17	7	3,3	170	9	47	72	15	36	12	37	430	2,1	0,86	23	210	35
Photinia x fraseri	NOV-23	30	10	2,5	12	230	11	28	59	49	20	6,7	420	16	27	59	54	120	18	76	1200	8,6	2,6	66	700	82
Elaeagnus ebbingei	NOV-23	4,3	1,5	0,38	2,1	88	1,6	4,3	13	6,3	2,8	1,4	74	2,8	24	26	9,2	16	4,6	13	190	1,5	0,41	9,4	170	15
Prunus laurocerasus	NOV-23	9,8	2,8	0,82	5,1	120	3,6	11	60	17	6	3,3	160	10	22	31	18	40	13	41	420	2,7	0,93	23	260	38
Geranium Rozanne	NOV-23	6,3	1,8	0,52	3,2	76	2,3	7	38	11	3,8	2,1	99	6,5	14	20	11	25	8,4	26	270	1,7	0,59	14	170	24
Drosanthemum hispidum	NOV-23	5,4	2,4	0,42	2,7	240	2,2	6,3	110	8,8	4,4	5,2	140	6	86	78	9,5	26	8,9	26	220	1,4	0,44	11	360	19
Pteris vittata	DEC-23	2,2	2,7	0,17	1	66	0,79	2,5	26	2,9	1,6	0,97	42	1,7	22	25	4,4	8,6	3,3	9,4	89	0,69	0,15	4,3	170	6,9
Viburnum lucidum	DEC-23	2,4	0,87	0,21	1	51	0,91	2,6	10	3,7	1,9	0,88	40	1,5	17	24	5	9,3	2,3	8,1	98	0,81	0,21	5,5	180	7,6
Buxus sempervirens	DEC-23	10	3,6	0,75	3,7	150	2,8	10	28	13	7,2	2,7	130	5,7	33	44	15	30	9,4	30	390	2,4	0,68	20	350	28
Nerium oleander	DEC-23	13	4	1,1	4,3	200	4,7	12	31	19	9	3,8	190	8	46	74	17	42	12	35	570	2,7	0,96	27	250	41
Photinia x fraseri	DEC-23	27	9	2,3	11	210	10	25	53	44	18	6,1	380	14	25	54	48	100	16	70	1000	7,8	2,3	59	630	74
Elaeagnus ebbingei	DEC-23	12	3,8	1	7,4	110	4,3	9,7	24	18	7,7	2,7	170	4,3	22	28	27	42	7,4	23	470	4	1	25	310	33
Prunus laurocerasus	DEC-23	13	3,7	1,1	5,3	110	4,6	12	58	20	8,1	3,5	160	8,3	24	33	23	47	10	34	510	3,7	1,1	28	310	42
Geranium Rozanne	DEC-23	7,6	2,2	0,63	3,2	69	2,8	7	35	12	4,9	2,1	98	5	14	20	14	28	6,3	21	310	2,2	0,66	17	190	25
Drosanthemum hispidum	DEC-23	6,2	2,6	0,51	3,2	240	2,4	7,4	110	10	4,8	5,2	150	6,6	80	74	11	28	11	31	260	1,6	0,51	13	380	23
Pteris vittata	JAN-24	1,4	2,2	0,11	0,7	53	0,5	1,8	22	2	1,1	0,69	32	1,3	17	19	2,8	6,3	2,7	7,7	57	0,44	0,11	2,9	110	4,9
Photinia x fraseri	JAN-24	23	7,7	1,9	9,2	180	8,5	22	46	38	16	5,2	320	12	22	47	42	88	14	61	890	6,7	2	50	540	63
Pteris vittata	MAR-24	3,8	4,8	0,29	1,7	120	1,4	4,4	47	5,1	2,8	1,7	73	3	39	43	7,5	15	5,9	17	150	1,2	0,26	7,4	290	12
Photinia x fraseri	MAR-24	38	13	3,2	15	300	14	36	76	62	26	8,6	530	20	36	77	69	140	24	100	1500	11	3,2	83	890	100
Pteris vittata	MAY-24	5	6,4	0,38	2,3	150	1,8	5,8	63	6,7	3,7	2,2	97	4	52	57	9,9	20	7,8	22	200	1,6	0,35	9,7	380	16
Photinia x fraseri	MAY-24	46	16	3,9	18	370	17	44	92	75	31	11	650	25	45	94	84	180	29	120	1800	13	4	100	1100	130
Pteris vittata	JUL-24	5,9	7,2	0,44	2,7	170	2,1	6,7	69	7,8	4,4	2,6	110	4,6	59	65	12	23	8,7	25	240	1,8	0,4	11	450	18
Photinia x fraseri	JUL-24	32	11	2,7	13	250	12	31	65	53	22	7,3	450	17	31	66	59	120	20	85	1300	9,4	2,8	71	760	89

Table S6. LOD, RSD (%) and elemental concentrations in fertilizer and fertigation solution

Element	LOD	RSD (%)	Fertilizer	Fertigation solution
**Al**	0.55 µg/L	20	0.11 mg/L	80 µg/L
**As**	0.15 µg/L	2.6	0.99 mg/L	16 µg/L
**Cr**	0.78 µg/L	6.0	0.52 µg/L	0.44 µg/L
**Cs**	0.001 µg/L	8.0	2.4 µg/L	0.17 µg/L
**Fe**	0.011 mg/L	14.0	2.3 mg/L	0.22 mg/L
**K**	0.044 mg/L	3.7	3700 mg/L	54 mg/L
**La**	0.001 µg/L	7.0	0.27 µg/L	0.001 µg/L
**Li**	0.012 µg/L	5.0	11 µg/L	1.7 µg/L
**Mg**	0.0023 mg/L	2.4	35 mg/L	21 mg/L
**Mn**	0.032 µg/L	9.6	6.5 mg/L	3.0 µg/L
**Mo**	0.010 µg/L	4.7	0.72 mg/L	12 µg/L
**Na**	0.068 mg/L	3.5	70 mg/L	6.7 mg/L
**Ni**	0.013 µg/L	5.0	46 µg/L	1.3 µg/L
**Pb**	0.006 µg/L	7.0	8.2 µg/L	0.14 µg/L
**Rb**	0.007 µg/L	3.6	0.84 mg/L	16 µg/L
**Sb**	0.007 µg/L	6.3	1.4 µg/L	0.55 µg/L
**Sn**	0.008 µg/L	13	0.25 µg/L	0.045 µg/L
**Ti**	0.056 µg/L	34	9.0 µg/L	0.089 µg/L
**Tl**	0.005 µg/L	10	1.4 µg/L	0.020 µg/L
**V**	0.25 µg/L	8.0	<LOD	1.2 µg/L
**Zn**	0.060 µg/L	18	14 mg/L	0.44 mg/L
**Zr**	0.060 µg/L	12	6.0 µg/L	0.12 µg/L

1.3 Weather surveys

A precise survey of weather conditions during the experiments can be found at https://it.weatherspark.com (https://it.weatherspark.com/m/71779/7/Condizioni-meteorologiche-medie-nel-mese-di-JULlio-a-Roma-Italia). In addition, a public survey of air pollution can be found at https://www.comune.roma.it) https://www.comune.roma.it/web/it/scheda-servizi.page?contentId=INF85868

Although the prevailing wind regime in Rome is generally dominated by western and south-western airflows, local particle dispersion and deposition are strongly influenced by urban morphology and traffic-induced turbulence, which can substantially affect microscale transport dynamics [6].

Table S7. Specific primers used for qRT-PCR analysis.

Name	Primer sequence 5'-3'	References
PvEF1b For	AGC TTG AAG AGG CTG TTC GA	7
PvEF1b Rev	ACA AGA TCG TCT TCG ATT GTC AT	
PvFeSOD For	TGA CGT GTG GGA GCA TGC CTA	This paper
PvFeSOD Rev	TGC CTT CGC CCG TTC CAG TC	
PvAPX For	CCG GAA GTC CAG GTG GCC AGT	
PvAPX Rev	AGC GCT GCA TCA GTT GGA AGA ACC	
PvCesA For	TCTCTGCTCACTTGTTTGCC	
PvCesA Rev	AGACATAAAGCTCACCAAAGTCC	
PvβGal8 For	CGTTATTGGCCCACTTATCTTGC	
PvβGal8 Rev	GGCACATGATACCTGCTTTGAG	
PfTHF For	CAGGATAAGAAGGTTGTTACTTGCC	8
PfTHF Rev	TGACTGTAACCACCACCTGC	
PfCeSA For	TTCCTTTTGGCTGAAGCCCG	
PfCeSA Rev	GCCAGAATCACAAGCCTGAAG	
PfAPX2 For	CTTTTACCAGCTGGCTGGAGTT	
PfAPX2 Rev	GACCTTCTGGTGGTGGTTCTTGT	
PfFe-SOD For	GGTGAAGAGTCCTAATGCCGT	
PfFe-SOD Rev	GTAAGCATGCTCCCAAACATCG	
PfβGal2 For	CATTGGCCGGGATACATTGC	
PfβGal2 Rev	ATCTCTGAGAAGGCTTGCCG	

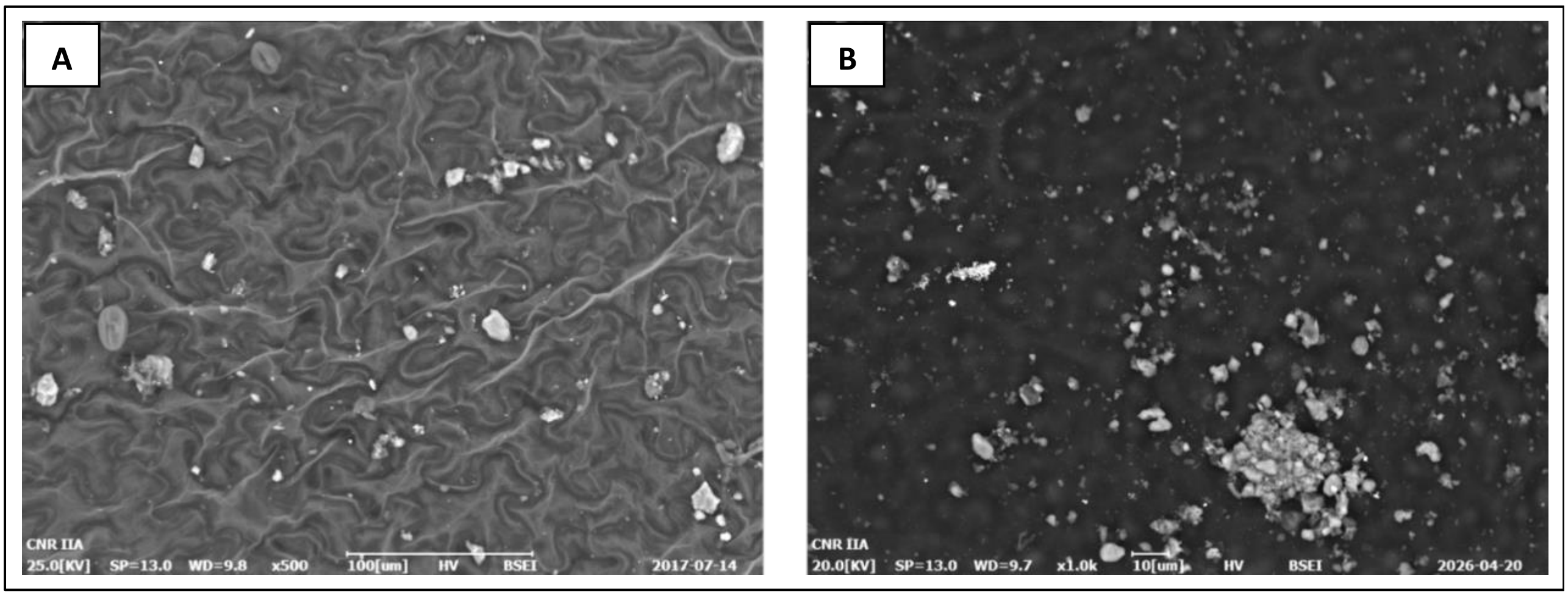

Figure S3. SEM micrographs (BSE-SEM images) showing fields of view of deposited PM onto non-washed *Pteris vittata* **(A)** and *Photinia × fraseri* **(B)** leaves. SEM conditions for imaging are visible in micrographs.

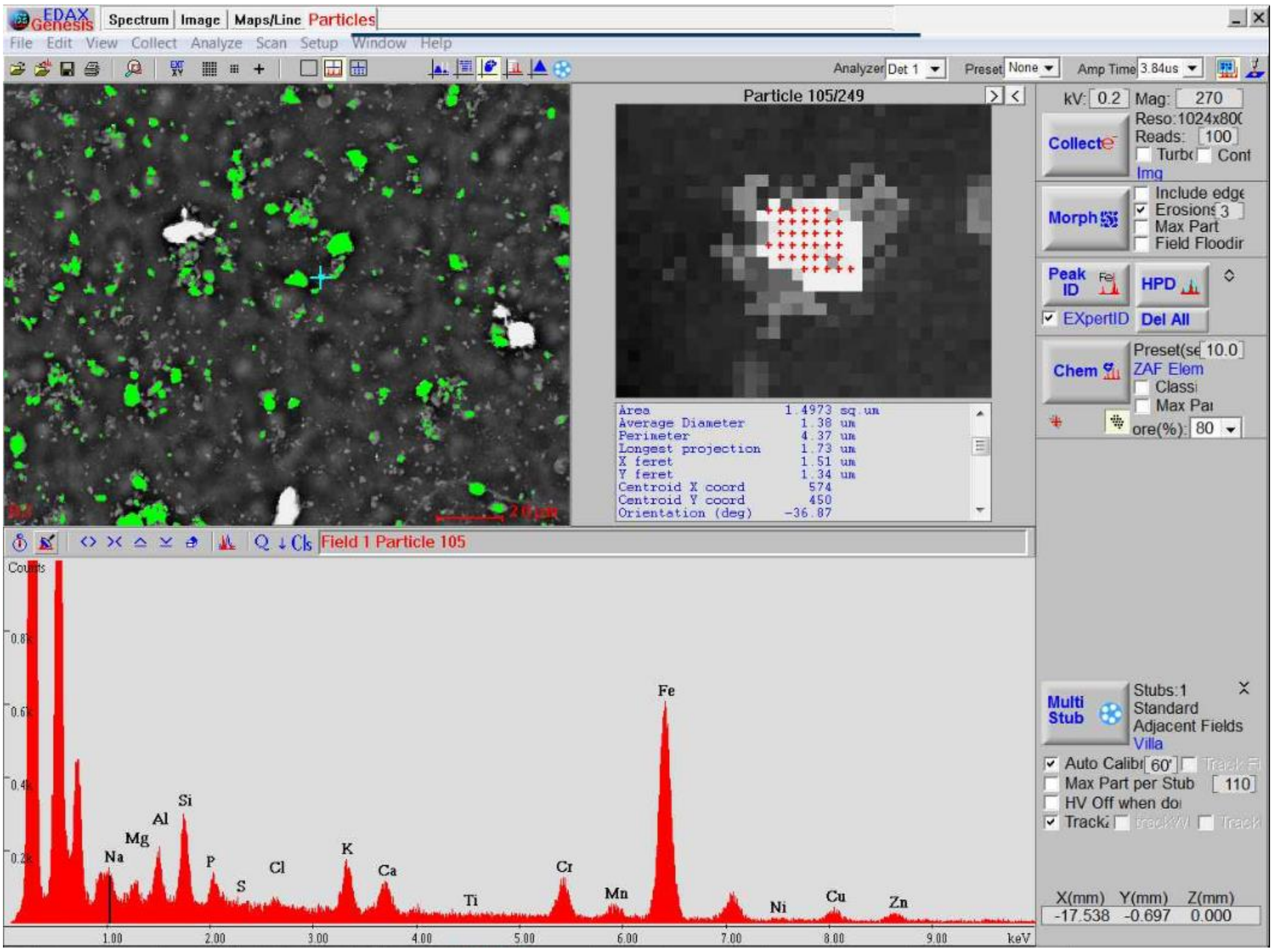

Figure S4. SEM particles field observed by automated microanalysis, evidencing a Fe particle in the non-washed *Photinia × fraseri* PM deposition.

Table S8. Descriptive statistics and temporal variability metrics used to assess the stability of elemental concentrations across the monitoring period. Mean, SD, Coefficient. Values are expressed as ng cm^{-2}, except for Al, Fe, K and Mg which are expressed as µg cm^{-2}

		Insoluble Fraction				Soluble Fraction				Leaf Fraction			
**Species**	**Element**	**CV%**	**Min**	**Max**	**Max/ Min**	**CV%**	**Min**	**Max**	**Max/ Min**	**CV%**	**Min**	**Max**	**Max/ Min**
P. x fraseri	**Al**	**22**	22.88	45.94	2.01	51	0.01	0.04	4.81	41	0.33	1.76	5.24
	**Fe**	**22**	21.71	43.66	2.01	27	0.03	0.06	2.07	51	0.56	3.51	6.28
	**K**	**22**	45.82	92.32	2.01	45	9.10	38.71	4.25	27	536.09	1170.96	2.18
	**Mg**	**22**	5.21	10.50	2.02	36	1.10	2.94	2.67	23	36.00	79.75	2.22
	**Cr**	**23**	180.67	365.01	2.02	31	0.73	1.99	2.71	58	5.72	36.52	6.38
	**Mn**	**22**	322.49	649.26	2.01	42	10.43	34.61	3.32	42	195.45	759.96	3.89
	**Ti**	**22**	887,54	1784.55	2.01	27	0.42	1.01	2.40	37	51.02	161.29	3.16
	**Zn**	**22**	539.54	1084.02	2.01	30	47.25	122.53	2.59	28	169.24	467.13	2.76
	**Cs**	**22**	8.50	17.06	2.01	57	0.06	0.41	7.18	29	2.06	4.75	2.30
	**Mo**	**22**	12.20	24.58	2.01	39	0.30	0.94	3.11	29	20.78	45.72	2.20
	**Rb**	**22**	87.76	176.16	2.01	54	24.54	99.51	4.06	32	235.85	762.13	3.23
	**Sb**	**24**	14.34	29.05	2.03	28	0.09	0.25	2.87	44	0.45	3.05	6.73
	**Sn**	**23**	60.76	122.92	2.02	32	0.05	0.20	3.72	61	1.05	8.63	8.24
P. vittata	**Al**	68	0.90	9.91	10.98	90	0.00	0.03	12.64	70	0.02	0.26	12.60
	**Fe**	65	1.33	10.83	8.16	//	<LOD	<LOD	//	52	0.12	0.56	4.75
	**K**	62	18.33	109.07	5.95	68	20.86	183.15	8.78	26	60.05	123.83	2.06
	**Mg**	62	0.48	4.19	8.65	73	0.24	4.86	19.88	35	1.54	7.33	4.76
	**Cr**	62	42.06	274.83	6.53	81	0.18	2.31	13.18	56	0.33	3.69	11.17
	**Mn**	64	24.08	176.55	7.33	81	1.54	37.09	24.08	58	7.00	99.06	14.16
	**Ti**	69	35.54	394.91	11.11	66	0.09	0.65	7.62	41	2.40	10.77	4.49
	**Zn**	70	63.02	746.62	11.85	50	17.90	155.13	8.67	47	20.46	122.22	5.97
	**Cs**	69	0.31	3.51	11.40	61	0.03	0.28	10.25	45	0.04	0.14	3.89
	**Mo**	64	0.99	7.35	7.41	80	0.06	1.87	29.34	42	0.82	5.71	6.99
	**Rb**	65	4.52	37.11	8.21	65	10.28	69.01	6.71	44	6.25	20.38	3.26
	**Sb**	62	2.18	13.76	6.32	44	0.02	0.11	5.04	71	0.02	0.47	24.48
	**Sn**	62	6.10	38.95	6.38	48	0.02	0.14	5.94	79	0.05	1.69	30.97

Table S9. Relative partitioning of selected PM non-exhaust traffic tracers among soluble, insoluble and leaf fractions in *Pteris vittata* and *Photinia x fraseri*, expressed as Soluble/Insoluble and Leaf/Insoluble concentration ratios.

Element	Species	Soluble/Insoluble	Leaf/Insoluble
**Al**	*Pteris vittata*	0.002	0.025
	Photinia x fraseri	0.001	0.035
**Ti**	*Pteris vittata*	0.002	0.039
	Photinia x fraseri	0.001	0.070
**Cr**	*Pteris vittata*	0.008	0.017
	Photinia x fraseri	0.005	0.077
**Sb**	*Pteris vittata*	0.012	0.033
	Photinia x fraseri	0.011	0.099
**Sn**	*Pteris vittata*	0.005	0.030
	Photinia x fraseri	0.002	0.044
**Rb**	*Pteris vittata*	1.878	0.683
	Photinia x fraseri	0.388	3.756
**Cs**	*Pteris vittata*	0.084	0.049
	Photinia x fraseri	0.016	0.254

Table S10. Relative partitioning of selected plant nutrient elements among soluble, insoluble and leaf fractions in *P. vittata* and *Photinia x fraseri*, expressed as Soluble/Insoluble and leaf/Insoluble concentration ratios.

Element	Species	Soluble/Insoluble	Leaf/Insoluble
**Fe**	*Pteris vittata*	0.002	0.060
	Photinia x fraseri	0.001	0.064
**Mn**	*Pteris vittata*	0.196	0.601
	Photinia x fraseri	0.041	1.102
**Zn**	*Pteris vittata*	0.250	0.221
	Photinia x fraseri	0.106	0.473
**Mo**	*Pteris vittata*	0.242	0.990
	Photinia x fraseri	0.033	1.688
**K**	*Pteris vittata*	1.739	1.688
	Photinia x fraseri	0.331	11.891
**Mg**	*Pteris vittata*	1.427	2.446
	Photinia x fraseri	0.259	7.793

References

(1) World Flora Online Consortium. *World Flora Online*. World Flora Online. https://www.worldfloraonline.org/ (accessed 2026-06-28).

(2) Zhang, L.; Rothfels, C. J.; Ebihara, A.; Schuettpelz, E.; Le Péchon, T.; Kamau, P.; He, H.; Zhou, X.; Prado, J.; Field, A.; Yatskievych, G.; Gao, X.; Zhang, L. A Global Plastid Phylogeny of the Brake Fern Genus *Pteris* (Pteridaceae) and Related Genera in the Pteridoideae. *Cladistics* **2015**, *31* (4), 406–423. https://doi.org/10.1111/cla.12094.

(3) Dirr, M. *Manual of Woody Landscape Plants: Their Identification, Ornamental Characteristics, Culture, Propagation and Uses*; Stipes Pub., 2009.

(4) Brickell, C.; Britain), R. H. S. (Great. *A-Z Encyclopedia of Garden Plants*; Dorling Kindersley Limited, 2016.

(5) Hartmann, H. E. K. Aizoaceae. In *Illustrated Handbook of Succulent Plants: Aizoaceae A-E*; Hartmann, H. E. K., Ed.; Springer Berlin Heidelberg: Berlin, Heidelberg, 2002; pp 9–268. https://doi.org/10.1007/978-3-642-56306-5_2.

(6) Cai, M.; Xin, Z.; Yu, X. Spatio-Temporal Variations in PM Leaf Deposition: A Meta-Analysis. *Environ. Pollut.* **2017**, *231*, 207–218. https://doi.org/10.1016/j.envpol.2017.07.105.

(7) Cesaro, P.; Cattaneo, C.; Bona, E.; Berta, G.; Cavaletto, M. The Arsenic Hyperaccumulating Pteris Vittata Expresses Two Arsenate Reductases. *Sci. Rep.* **2015**, *5* (1), 14525. https://doi.org/10.1038/srep14525.

(8) Vergata, C.; Contaldi, F.; Baccelli, I.; Basso, M. F.; Santini, A.; Pecori, F.; Buti, M.; Mengoni, A.; Vaccaro, F.; Moura, B. B.; Ferrini, F.; Martinelli, F. How Does Particulate Matter Affect Plant Transcriptome and Microbiome? *Environ. Exp. Bot.* **2023**, *209*, 105313. https://doi.org/10.1016/j.envexpbot.2023.105313.
